# Fluorinated Boron-Dipyrromethene (BODIPY) Dyes: Bright and Versatile Probes for Surface Analysis

**DOI:** 10.1002/open.201200039

**Published:** 2013-01-09

**Authors:** Mandy Hecht, Tobias Fischer, Paul Dietrich, Werner Kraus, Ana B Descalzo, Wolfgang E S Unger, Knut Rurack

**Affiliations:** [a] Division 1.9 Sensor Materials, BAM Federal Institute for Materials Research and TestingRichard-Willstätter-Str. 11, 12489 Berlin (Germany) E-mail: knut.rurack@bam.de; [b] Division 6.8 Surface Analysis and Interfacial Chemistry, BAM Federal Institute for Materials Research and TestingUnter den Eichen 44–46, 12203 Berlin (Germany); [c] Division 1.3 Structural Analysis, BAM Federal Institute for Materials Research and TestingRichard-Willstätter-Str. 11, 12489 Berlin (Germany)

**Keywords:** amino groups, dyes, fluorescence, surface analysis, X-ray photoelectron spectroscopy

## Abstract

A family of bright boron-dipyrromethene-type fluorophores with a high number of fluorine atoms (F-BODIPYs) has been developed and characterized by X-ray crystallography and optical spectroscopy. The introduction of 3,5-bis(trifluoromethyl)phenyl and pentafluorophenyl moieties significantly enhances the photostability of such dyes, yielding for instance photostable near-infrared (NIR) fluorophores that show emission maxima>750 nm, when the BODIPY’s π system is extended with two (dimethylamino)styryl and (dimethylamino)naphthastyryl moieties, or green-emitting BODIPYs with fluorescence quantum yields of unity. When equipped with a suitable group that selectively reacts for instance with amines, F-BODIPYs can be used as potent dual labels for the quantification of primary amino groups on surfaces by X-ray photoelectron spectroscopy (XPS) and fluorescence, two powerful yet complementary tools for the analysis of organic surface functional groups. The advantage of reactive F-BODIPYs is that they allow a fast and non-destructive mapping of the labelled supports with conventional fluorescence scanners and a subsequent quantification of selected areas of the same sample by the potentially traceable XPS technique. The performance is exemplarily shown here for the assessment of the amino group density on SiO_2_ supports, one of the most common reactive silica supports, in particular, for standard microarray applications.

## Introduction

Tailor-made surfaces expressing a homogenous distribution of a certain type of functional group, such as, alcohol, carboxyl, primary amine, thiol, aldehyde or epoxy, are of paramount importance in many areas of the (bio)chemical sciences. For instance, such surfaces are essential in many bioanalytical or diagnostic applications, because these primary functional groups can be used for further attachment of biomolecules, such as, oligonucleotides, peptides, sugars, antibodies or enzymes, rendering them suitable for microarray-based assays or as sensory layers.^1^–^5^ The efficiency of the immobilization process and the quality of the final biochemically functionalized surface strongly depend on the density and, even more importantly, on the accessibility of the surface functional groups.^6^ For the reliable characterization of the chemical nature and surface concentration of functional groups, robust and fast analytical tools are urgently required. Several techniques have for instance been described to determine the density of primary amino groups on solid surfaces—many of the respective publications deal with the characterization of amino groups on polymer surfaces.^7^ Besides infrared spectroscopic techniques such as Fourier transform IR (FTIR, especially for thin films)^8^ and time-of-flight secondary ion mass spectrometry (ToF-SIMS),^9^ X-ray photoelectron spectroscopy (XPS) is one of the most widely used methods. However, XPS often shows inferior performance at low concentrations of surface functionalities and is not suitable for a direct identification and quantification of amino groups on surfaces, when these groups coexist with a manifold of other nitrogen-containing species with similar chemical shifts.^10^–^12^ Therefore, protocols have been developed in which the amino groups are labelled with molecular entities containing elements that are originally not present on the surface of the samples, for example, fluorine. After the labelling reaction, the concentration of this new element is assessed by XPS analysis and directly relates to the concentration of the particular functional group on the surface. Up to now, the reagents mostly used for amino-group labelling in XPS analysis are pentafluorobenzaldehyde (PFB),^13^, ^14^ (4-trifluoromethyl)benzaldehyde (TFBA),^15^, ^16^ 3,5-bis(trifluoromethyl)phenylisocyanate^17^ and trifluoroacetic anhydride (TFAA).^18^, ^19^ Other nitrogen-containing functional groups such as amides, imines and nitriles do not react with these derivatization reagents. However, an intrinsic drawback of XPS is that the limit of detection for a certain element lies typically at 0.1–1.0 atom- % (at- %),^20^ with all the atoms of label, functional group and bulk within the spot of irradiation counting in. To overcome these quantification difficulties of XPS at low labelling concentrations, optical methods in combination with an adequate labelling technique have been increasingly employed. Here, both colorimetric^21^–^23^ and fluorometric^24^–^27^ methods have been successfully used for surface-amino-group determination, with the second technique showing distinctly higher sensitivities. Dansyl hydrazine and pyrylium dyes are examples of possible fluorescent markers.^21^–^23^, ^26^ The amino-reactive compound fluram (4-phenylspiro-[furan-2(3 *H*),10-pthalan]-3,30-dione), which reacts with nucleophiles in general but forms a fluorescent product only with primary amines, was successfully employed for various surfaces.^28^, ^29^ However, translating the signal detected to an absolute number of functional groups is not trivial. Nonspecific adsorption and binding can result in enhanced background fluorescence and quenching phenomena. In addition, whereas XPS measurements can provide quantitative results that are traceable to a primary standard,^30^, ^31^ absolute fluorescence measurements are an intrinsically difficult task already for ideally dilute solutions,^32^ and most fluorescence techniques only provide relative results. On the other hand, fluorescence scanning techniques are much faster, non-destructive and technically as well as methodologically much simpler than XPS. For traceable and quantitative yet rapid surface chemical analysis, the quest is thus to find a way to directly link both methods with their unique features.

Boron-dipyrromethene (BODIPY) dyes have up to now only been rarely used for fluorescence labelling of surface species,^25^ despite their popularity as functional dyes, and the tremendous progress in the BODIPY field during the past ten years.^33^–^39^ Moreover, the remarkable versatility of BODIPYs has sparked intense research on new modification strategies to enable their attachment to biological substrates and also to tune their optical properties. Today, BODIPYs are widely used as biomolecule markers, fluorescent switches, chemosensors and laser dyes.^33^–^38^ Several new strategies for their functionalization have recently been developed, among which halogenation of the BODIPY core is particularly interesting because the introduction of a halogen atom to the dipyrrin core facilitates further derivatization through aromatic nucleophilic substitution and palladium-catalyzed coupling reactions.^40^, ^41^ The most important challenges in recent BODIPY chemistry perhaps include the development of dyes with longer-wavelength absorption and emission profiles, and the preparation of dyes with additional functional groups for covalent attachment.

**Scheme 1:**
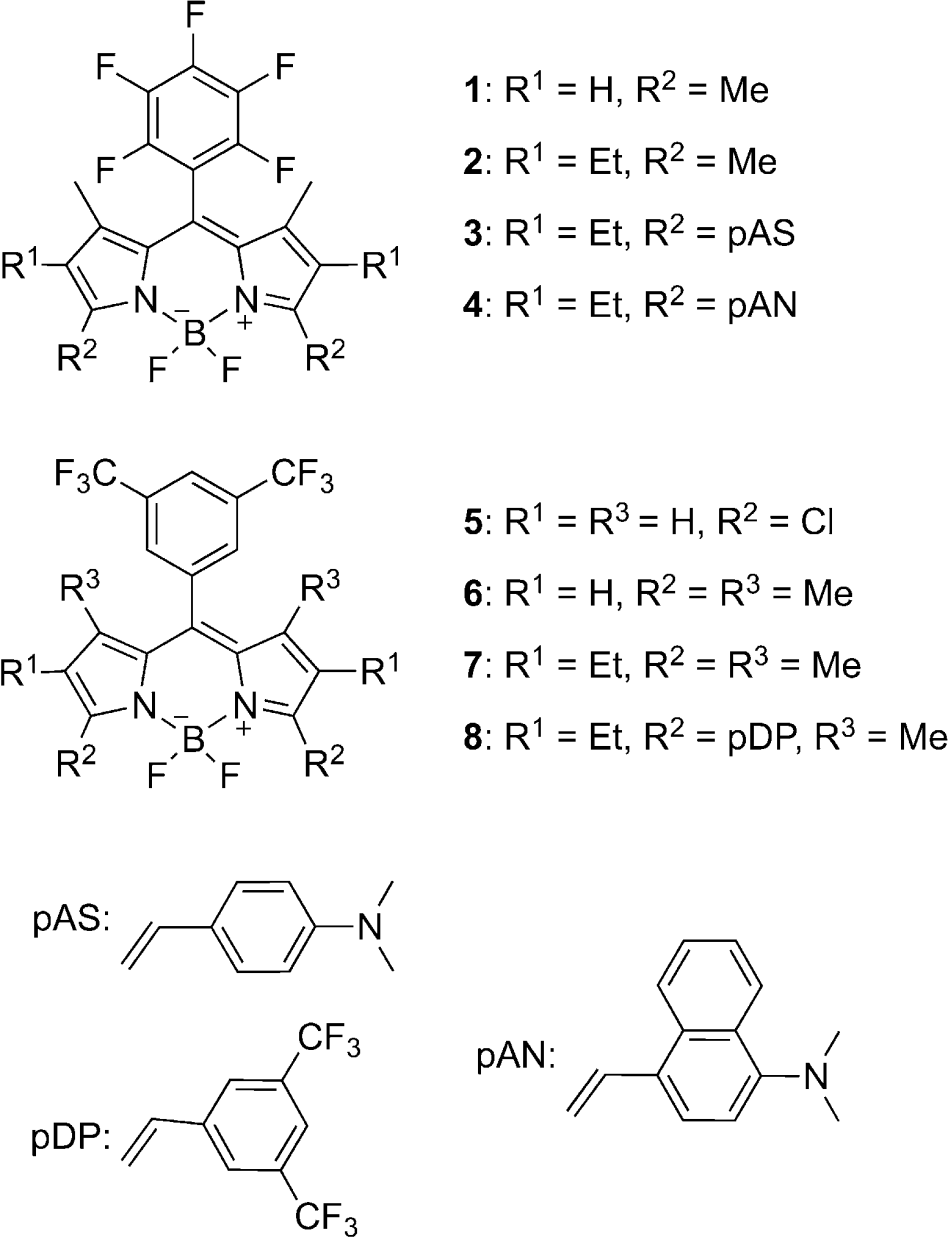
Chemical structures of the compounds investigated in this work.

Based on our experience in the field of BODIPY-dye chemistry^42^–^45^ and being increasingly confronted with the request for photo- and chemically stable dyes for reliable labelling purposes as well as high-performance dyes for the near infrared (NIR) region, we embarked on the design, synthesis, characterization and application of BODIPY dyes containing a high amount of fluorine atoms.^46^–^48^ Regarding the comparability of fluorescence and XPS measurements, we reasoned that highly fluorinated BODIPY dyes might combine several advantageous features, that is, high stability, prominent spectroscopic properties and considerably low limits of detection for a unique XPS-suitable atom such as fluorine. Here, we describe the synthesis and characterization of a series of novel fluorine-containing BODIPY-type fluorophores and labelling agents (Scheme 1). In addition, some of the BODIPYs were functionalized appropriately via the Knoevenagel condensation to generate styryl-substituted NIR-emitting fluorophores. The performance of selected dyes as dual XPS/fluorescence labels is studied, providing detailed results on the chemical composition and distribution of the immobilized fluorescence labels on aminated SiO_2_ supports.

## Results and Discussion

Fluorine substitution is generally considered as rendering a certain dye considerably more photostable than its hydrogen-containing analogue, often also improving its spectroscopic characteristics.^49^–^51^ For XPS, on the other hand, it is essential to increase the amount of a certain marker atom on a label as much as possible to achieve better limits of detection. Thus, the task for a dual XPS/fluorescence label is to increase the number of fluorine atoms on a dye while still allowing further attachment of reactive groups or its conjugation to surface functional groups. Whereas highly fluorinated cyanine dyes are virtually nonexistent, fluorinated rhodamine and fluorescein dyes have been reported, yet either their fluorine content is not significantly high or the possibilities for further functionalization are limited and synthesis can be tedious.

**Scheme 2:**
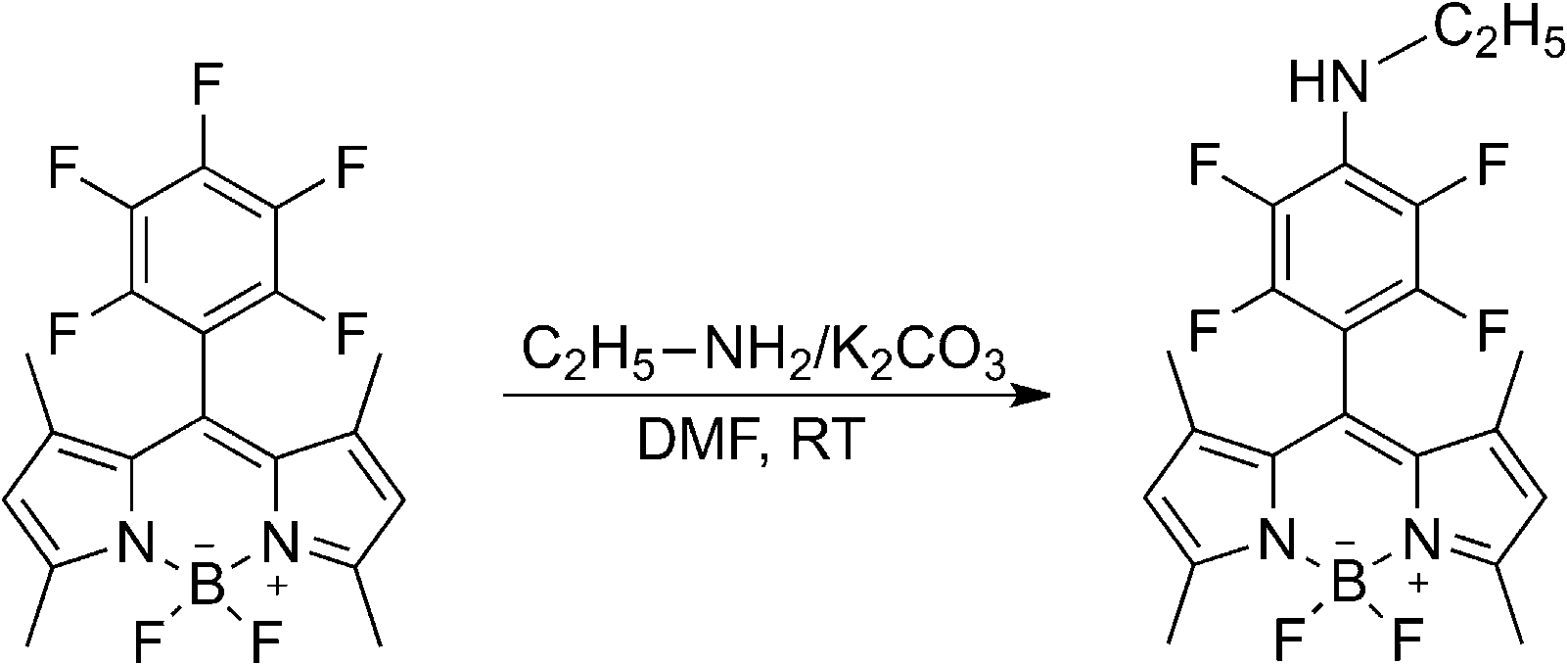
Substitution reaction at the pentafluorophenyl moiety observed by Vives et al.^52^

Based on our own work on fluorinated BODIPYs,^46^–^48^ we were intrigued by recent findings of G. Vives et al. that a dye such as **1**, which carries a significant amount of fluorine atoms (16.2 at- %), reacts with thiols and amines in solution because the *p*-fluoro atom is a good leaving group for a nucleophilic aromatic substitution reaction (Scheme 2);^52^ and we reasoned that such pentafluorophenyl-substituted BODIPYs might be good candidates for our purposes of developing dual XPS/fluorescence labels for surface amino groups.

Moreover, besides the facility of fluorine atom introduction, especially the modularity of BODIPY dyes with the unique features of using the *meso*- and for instance the 3,5-positions independently for functionalization would allow a facile tuning of either the fluorine content, the spectral region of absorption and fluorescence or both by attaching selected substituents to the 3,5-positions. We thus developed a series of fluorinated BODIPYs **1**–**8** (Scheme 1). The fluorine content of these BODIPYs increases from 12.7 to 21.6 at- % in the order **2**, **7**, **1**, **6**, **8** and **5**; for comparison, the conventional XPS labels mentioned above possess a fluorine content between 17.6 and 46.1 at- %. Although **8** presents a long-wavelength dye with an exceptionally high fluorine content, **3** and **4** despite their lower fluorine content also promise to render very photostable NIR dyes due to their architecture.

### Synthesis

The newly synthesized dyes **6** and **7** were obtained in 19 and 45 % yield, respectively, by condensation of the corresponding aldehyde with 3-ethyl-2,4-dimethylpyrrole (or 2,4-dimethylpyrrole, respectively) in the presence of trifluoroacetic acid (TFA), followed by oxidation with *p*-chloranil, deprotonation with di-*iso*-propylethylamine (DIPEA) and complexation with BF_3_**⋅**OEt_2_. Following this synthetic route, the yields of **1** and **2** were increased compared with literature reports (25 and 55 %, respectively).^53^, ^54^ Using the mild oxidation agent *p*-chloranil instead of previously used 2,3-dichloro-5,6-dicyanobenzoquinone (DDQ),^46^–^48^ the formation of side products can be reduced, rendering improved yields. Moreover, a drop-wise addition of *p*-chloranil can apparently slightly increase the yield of **1** even more, as reported by Vives et al.^52^ All compounds yielded crystals suitable for X-ray structural analysis (see below).

**Scheme 3:**
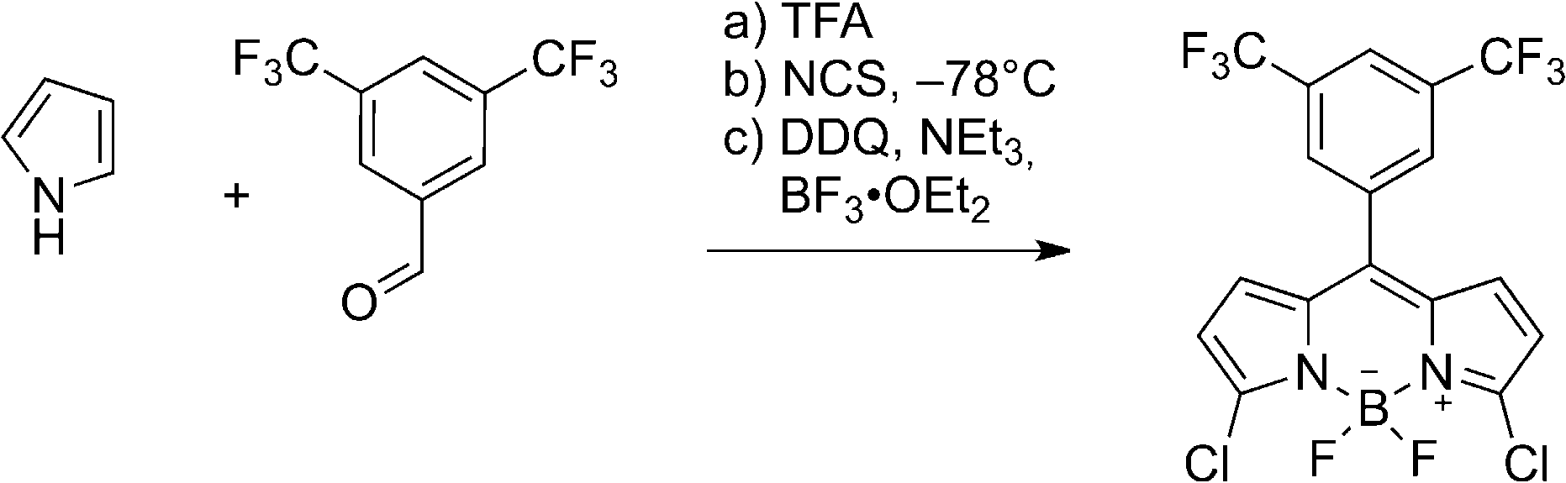
Synthesis of **5** via a 3-step route. *Reagents and conditions*: a) TFA; b) NCS, −78 °C; c) DDQ, NEt_3_ followed by BF_3_**⋅**OEt_2_.

The most common approach to introduce functional groups at 3- and 5-positions of the BODIPY core involves the synthesis of appropriately substituted pyrroles, which in turn requires the often time-consuming synthesis of (photo)chemically rather labile pyrrole precursors. Recent synthetic developments in BODIPY chemistry thus focused on simpler methods such as the introduction of chloro substituents at the 3,5-positions and their subsequent nucleophilic substitution. The substitution reaction conditions can be adjusted in such a manner that either mono- or disubstitution occurs. Studies in solution showed that at room temperature only one chlorine atom is substituted. The reaction of both chlorine functionalities can be achieved under more drastic conditions, that is, increased reaction temperature or addition of a base. As shown in Scheme 3, **5** with two amino-reactive chloro atoms could be obtained in a three-step synthesis, including chlorination prior to oxidation and complexation.^55^–^57^

Separation of the intermediates from numerous side products after each step is advantageous over the common BODIPY one-step procedure. The π-extended mono- and distyryl-BODIPY derivatives **3**, **4** and **8** were prepared by a Knoevenagel-type condensation in the presence of piperidinium acetate as catalyst, together with a small amount of activated molecular sieves to adsorb the water produced during the reaction. For **8**, a stoichiometric amount of dimethylformamide (DMF) had to be used with dichloromethane to afford the condensation of **7** with the electron-deficient 3,5-bis(trifluorormethyl)benzaldehyde. The desired product could not be obtained in toluene. Except for **4**, products resulting from the single condensation reactions were only present in traces and could be easily separated by column chromatography. The structures of the new compounds were confirmed by ^1^H and ^13^C NMR spectroscopy, HRMS as well as X-Ray structure analysis, in case appropriate crystals could be obtained.

### X-ray structure analysis

**Figure 1:**
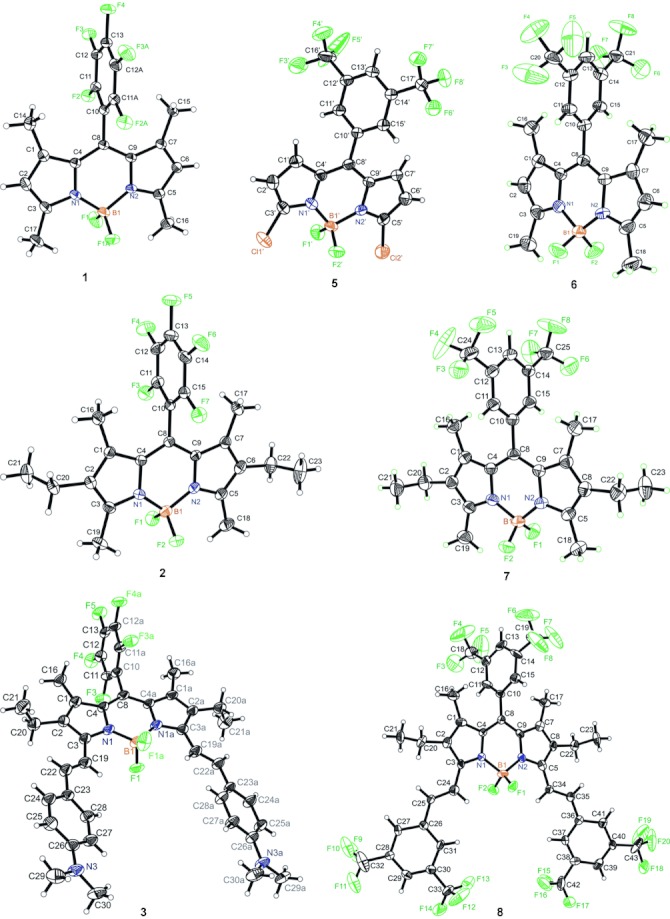
*Ortep* representation of the compounds investigated by X-ray structure analysis; atomic labelling shown with 30 % probability displacement ellipsoids.

The molecular structures of BODIPYs **1**–**3** and **5**–**8** were determined by single-crystal diffraction analysis, and the corresponding molecular configurations are shown in Figure 1. Selected crystallographic data and structure refinement parameters are listed in Table 1. The observed geometric parameters of all crystal structures are generally comparable with data previously reported for other BODIPY-based compounds.^58^, ^59^ For comparison, the known structure of **1**^60^ was solved by us again. All compounds crystallize in a monoclinic or orthorhombic space group with similar crystal packing. Except for **5**, no significant intermolecular interactions such as hydrogen bonds (H bonds) or π–π interactions are present in the crystal lattices of the dyes investigated. Only weak π–π interaction was found for **6** with a plane-to-plane distance of 4.24 Å between the two pyrrole rings from the BODIPY core. The BODIPY skeleton formed by three conjugated heterocyclic rings is almost planar, with an rms deviation ranging from 0.0126 Å (for **3**) to 0.0659 Å (for **8**; see Table 2).

**Table 1:** Selected crystallographic data and structure refinement parameters for 2, 3, and 5–8

Compd No.	2	3	5	6	7	8
Chemical formula	C_23_H_22_BF_7_N_2_	C_41_H_40_BF_7_N_4_	C_17_H_7_BCl_2_F_8_N_2_	C_21_H_17_BF_8_N_2_	C_25_H_25_BF_8_N_2_	C_43_H_29_BF_20_N_2_
Formula Mass [g mol^−1^]	470.24	732.58	472.96	460.18	516.28	964.49
Crystal system	monoclinic	monoclinic	monoclinic	orthorhombic	monoclinic	orthorhombic
Space group	*P*2_1_*/c*	*C*2*/c*	*P*2_1_*/c*	*Pca*2_1_	*P*2_1_*/n*	*Pna*2_1_
*a* [*Å*]	8.7452(7)	16.947(5)	23.127(3)	25.222(4)	8.915(3)	*28.715(4)*
*b* [*Å*]	11.6694(8)	27.710(6)	9.3301(10)	11.8719(16)	11.515(4)	17.658(3)
*c* [*Å*]	22.5946(14)	7.8970(19)	17.4917(18)	6.9173(12)	24.369(9)	8.6785(12)
*α* [°]	90.00	90.00	90.00	90.00	90.00	90
*β* [°]	95.630(4)	94.460(15)	91.269(7)	90.00	96.699(6)	90
*γ* [°]	90.00	90.00	90.00	90.00	90.00	90
Unit cell volume [Å^3^]	2294.7(3)	3697.3(16)	3773.4(7)	2071.3(6)	2484.5(16)	4400.5(11)
Temperature [K]	296(2)	296(2)	296(2)	296(2)	296(2)	296(2)
No. of formula units per unit cell, *Z*	4	4	8	4	4	4
Radiation type	Mo-Kα	Mo-Kα	Mo-Kα	Mo-Kα	Mo-Kα	Mo-Kα
Absorption coefficient [*μ* mm^−1^]	0.119	0.102	0.427	0.137	0.123	0.144
No. of reflections measured	4536	14 273	42 910	21 906	20 785	54 364
No. of independent reflections	4536	4518	9330	5011	6166	10 722
*R_int_*	0.0000	0.1155	0.0995	0.1394	0.0552	0.1840
Final *R_1_* values (*I*>2*σ*(*I*))	0.0739	0.0544	0.0729	0.0840	0.0688	0.0690
Final *wR*(*F*^2^) values (*I*>2*σ*(*I*))	0.1832	0.1027	0.1969	0.2068	0.2026	0.1257
Final *R_1_* values (all data)	0.1620	0.2418	0.1673	0.1765	0.1397	0.3300
Final *wR*(*F*^2^) values (all data)	0.2138	0.1290	0.2257	0.2390	0.2327	0.1672
Goodness of fit on *F*^2^	1.073	0.826	0.795	0.815	0.950	0.628
CCDC number	89 3402	89 3400	89 3403	89 3401	89 3399	90 5368

**Table 2:** Dihedral angle between dipyrrin core and *meso*-substituent at C8 (*Θ*_dp–meso_) and rms deviation of the BODIPY core

Compd	*Θ*_dp–meso_[a] [°]	rms deviation of dp[b] [Å]
**2**	89.83 (0.10)	0.0289
**3**	81.61 (0.08)	0.0126
**5**	59.28 (0.10) −59.43 (0.09)	0.0518 (for C1……) 0.0297 (for C1′……)
**6**	81.03 (0.09)	0.0257
**7**	81.05 (0.08)	0.0224
**8**	76.14 (0.16)	0.0659

[a] Planes defined by atoms C1–C9,N1,N2,B1 for dp and C10–C15 (or C10′–C15′ or C10,C11,C11a,C12,C12a,C13) for substituent in *meso*-position, see Figure 1.

[b] dp=BODIPY core, see footnote [a].

**Figure 2:**
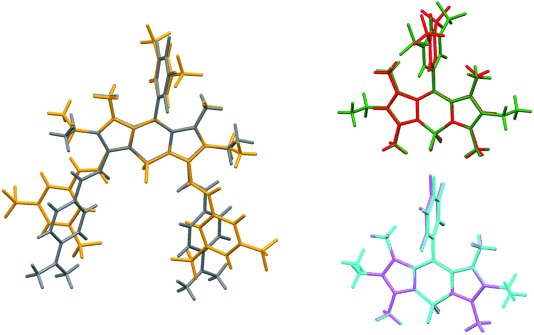
Overlay of **3** (grey) and **8** (yellow), **6** (red) and **7** (green), and **1** (cyan) and **2** (magenta).

In all cases, the boron atom has a slightly distorted tetrahedral coordination with the two fluorine atoms being perpendicularly oriented with respect to the dipyrrin plane. The average B–N bond length amounts to 1.557(5) Å, indicating that all compounds possess single B–N bonds. The two B–N distances are virtually identical, implying the expected delocalisation of the positive charge. The average B–F bond length is 1.390(5) Å and the average N–B–N and F–B–F angles are 107.2(3)° and 110.3(3)°, respectively. The biggest difference in the B–F bond lengths is found for **8** with 1.369(8) and 1.424(8) Å (see Table S1 for detailed data). BODIPY **8** also possesses the largest rms of the BODIPY core with 0.066 Å and an angle of 8.9(4)° between the two pyrrole rings. The angle between the two phenyl rings of the styryl extensions in the 3,5-positions is 8.2(4)°, much larger than that between the respective groups in **3** with only 0.5(8)°, indicating virtual parallel arrangement of the styryl moieties in the latter. However, whereas the styryl extensions and B–F bond lengths in **3** are more similar, the average angle between the styryl extensions and the BODIPY core is significantly smaller in **8** (25.7°) compared with **3** (33.0°). This overall higher planarity of the bis-styryl-BODIPY skeleton in **8** leads to a propeller-like distortion of the BODIPY core,^44^ entailing the observed differences. Due to steric repulsion from the methyl groups attached to C1 and C7, the (phenyl or pentafluorophenyl) ring appended to C8 is strongly twisted out of the BODIPY mean plane, with dihedral angles ranging from 76.1(2)° to 89.8(1)° (Table 2). An overlay of the respective molecular configurations shows the differences of the molecular conformations, respectively, and the different arrangements of the substituents (Figure 2). The structures of **3** and **8** show the greatest differences, presumably because of the features discussed above.

**Figure 3:**
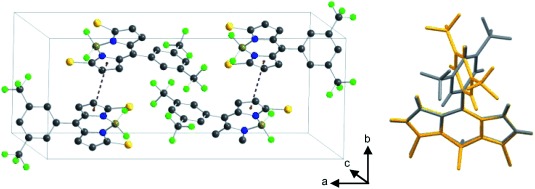
Crystal packing of **5** and overlay of two symmetry independent molecules, red dotted lines showing π–π electron interactions.

In **1**, the BODIPY core lies on a crystallographic mirror plane that bisects the BF_2_ and pentafluorophenyl groups. The dihedral angle between the pentafluorophenyl ring and the tricyclic system is thus 90° by symmetry. The asymmetric unit of **3** consists of half a molecule. For symmetry reasons, the two phenyl rings in the compound are parallel and the BODIPY core has the lowest rms deviation of 0.0126 Å. The asymmetric unit of **5** contains two different molecules. Due to the lack of substituents at positions 1 and 7, the dihedral angle, *θ*_dp–meso_, between the phenyl ring at C8 and the dipyrrin plane amounts to only 59.28°/−59.43°, respectively. Figure 3 shows a superposition of these two molecules. The molecules are arranged in a head-to-tail fashion in the unit cell. The two *meso*-phenyl rings of neighbouring molecules are virtually parallel (Figure 3). All compounds with CF_3_ groups exhibit large displacement ellipsoids for the F atoms, in particular **8**, presumably because they are either disordered or slightly moveable due to missing interactions in the crystal lattice.

### Optical spectroscopic properties

**Figure 4:**
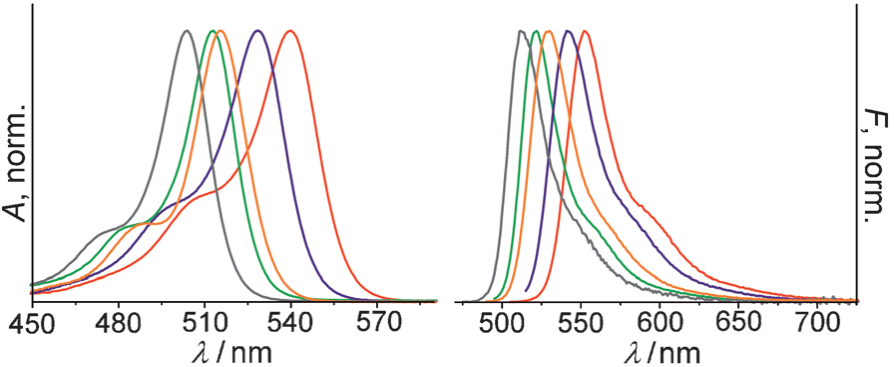
Absorption and emission spectra of **1** (**—**), **2** (**—**), **5** (**—**), **6** (**—**) and **7** (**—**) in MeCN.

**Figure 5:**
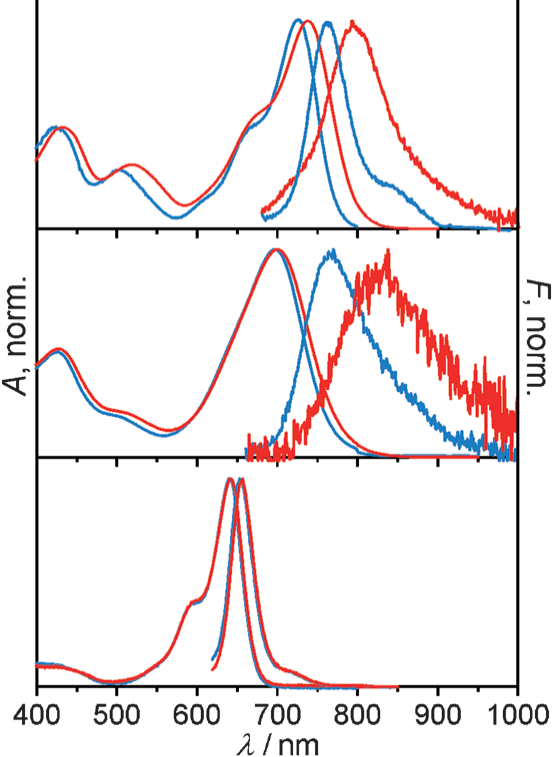
Absorption and fluorescence spectra of **3** (top), **4** (middle) and **8** (bottom) in Bu_2_O (**—**) and THF (**—**).

Table 3 shows the spectroscopic features of the compounds investigated by absorption as well as steady-state and time-resolved fluorescence spectroscopy in various solvents with increasing polarity from hexane to methanol. For all dyes, except **3** and **4**, the maxima of the absorption and fluorescence spectra are essentially solvent independent (Table 3), indicating the relatively small change in dipole moment between the ground and the excited state of these compounds.^61^–^64^ BODIPYs **1**–**8** possess the characteristic spectroscopic features of BODIPYs including narrow spectral bands with two absorption maxima, an intense S_0_–S_1_ transition and a weak shoulder at the high-energy side due to the 0–1 vibrational transition, an emission band of mirror-image shape and mono-exponential fluorescence decay kinetics.^61^, ^65^–^67^ Figure 4 and 5 illustrate the spectral features of the dyes investigated.

**Table 3:** Selected spectroscopic data of 1–8 in various solvents at 298 K (for additional data, see Table S2)

Compd	Solvent[a]	*λ*_abs_ [nm]	*λ*_em_ [nm]	Δ 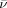 _abs−em_ [cm^−1^]	*Φ*_f_	*τ*_f_ [ns]	*k*_r_ ×10^8^ [s^−1^][b]	*k*_nr_ ×10^8^ [s^−1^][b]
**1**	Hex	517	525	295	1.00	5.74	1.8	0.0
	Et_2_O	516	523	332	1.00	6.01	1.8	0.0
	THF	517	526	331	1.00	5.53	2.0	0.0
	MeCN	513[c]	521	373	1.00	6.05	1.7	0.0
**2**	Hex	542	554	400	0.94	6.45	1.5	0.1
	Et_2_O	541	554	401	1.00	6.72	1.5	0.0
	THF	543	555	398	0.93	6.18	1.5	0.1
	MeCN	539[d]	553	470	0.85	6.74	1.3	0.2
**3**	Bu_2_O[e]	723	762	776	0.29	1.67	1.7	4.3
	Et_2_O	723	772	978	0.28	1.51	1.9	4.8
	THF	737	798	1084	0.15	0.94	1.6	9.1
	MeCN	739[f]	849	1998	0.05	0.43	1.2	22.1
**4**	Hex	692	743	1010	0.23	1.39	1.6	5.5
	Et_2_O	694	780	1670	0.13	0.88	1.5	9.9
	THF	699	(830)	2373	0.06	0.40	1.5	23.5
**5**	Hex	521	536	537	0.12	0.70	1.7	12.6
	Et_2_O	518	532	578	0.11	0.71	1.6	12.5
	THF	518	533	648	0.19	1.09	1.7	7.4
	MeCN	515[g]	530	550	0.19	1.25	1.5	6.5
**6**	Hex	506	516	383	0.41	2.05	2.0	2.9
	Et_2_O	505	514	347	0.47	2.41	1.9	1.8
	THF	506	517	420	0.68	3.08	2.2	1.0
	MeCN	503[h]	513	388	0.62	3.40	1.8	1.1
**7**	Hex	531	544	484	0.59	4.13	1.4	1.0
	Et_2_O	529	543	487	0.65	4.71	1.4	0.7
	THF	530	543	486	0.76	4.73	1.6	0.5
	MeCN	528[i]	541	523	0.72	5.29	1.4	0.5
**8**	Hex	638	651	313	0.79	4.83	1.6	0.4
	Et_2_O	637	651	338	0.79	4.93	1.6	0.4
	THF	641	657	380	0.70	4.48	1.6	0.7
	MeCN	637[j]	650	338	0.80	4.96	1.6	0.4

[a] Hex=*n*-hexane, Bu_2_O=dibutyl ether, Et_2_O=diethyl ether, THF=tetrahydrofuran, MeCN=acetonitrile.

[b] *k*_r_=*Φ*_f_×*τ*_f_^−1^, *k*_nr_=(1−*Φ*_f_)×*τ*_f_^−1^; measurement uncertainties: ±0.01×10^8^ s^−1^.

[c] *ε*_*λ*abs_=76 660±4200 m^−1^ cm^−1^.

[d] *ε*_*λ*abs_=67 300±1160 m^−1^ cm^−1^.

[e] **3** is insoluble in hexane.

[f] *ε*_*λ*abs_=75 220±4190 m^−1^ cm^−1^.

[g] *ε*_*λ*abs_=78 230±1550 m^−1^ cm^−1^.

[h] *ε*_*λ*abs_=79 470±2040 m^−1^ cm^−1^.

[i] *ε*_*λ*abs_=56 500±1080 m^−1^ cm^−1^.

[j] *ε*_*λ*abs_=70 500±800 m^−1^ cm^−1^.

**Scheme 4:**
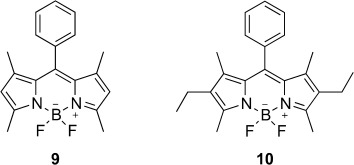
Model compounds **9**^65^ and **10**^43^ from the literature.

Electrostatic effects such as an enhanced dipole moment of the 1,3,5,7-tetramethyl compared with the 3,5-dimethyl and the 1,3,5,7-tetramethyl-2,6-diethyl BODIPY core lead to a more ionic nature of the 1,3,5,7-tetramethyl core and slightly blue-shifted absorption bands of **1** and **6** compared with **2**, **5** and **7**.^68^ When comparing the positions of absorption and fluorescence maxima for **6** and **1** as well as **7** and **2**, only a slight spectral shift occurs through the variation of the substitution pattern at the *meso*-phenyl group from 3,5-bis(trifluoromethyl)phenyl to pentafluorophenyl, presumably due to stronger electron withdrawing characteristics of the pentafluorophenyl moiety, which is in line with recent observations on the influence of such substituents in metal complex-catalyzed hydroamination reactions.^69^ The fluorescence quantum yields, *Φ*_f_, on the other hand are strongly affected by the *meso*-substituent. In the case of **1**, the value of *Φ*_f_ is virtually 1 for all solvents, whereas *Φ*_f_ of **6** varies around 0.6, a value that has also been found for the *meso*-phenyl analogue of **1**, compound **9** (Scheme 4).^65^ This can be explained by the stronger nonradiative decay due to the rotation of the trifluoromethyl groups and the strongly restricted rotation of the pentafluorophenyl moiety by the *o*-fluorine atoms in addition to the 1,7-methyl groups, as supported by the distinctly higher nonradiative decay rates (*k_nr_*) of **6**. The crystal structures show almost orthogonal orientation of the phenyl substituent for all dyes bearing 1,7-methyl groups. In addition, also for the hexa-alkyl-substituted BODIPY core, an increased fluorescence quantum yield is found, when it is equipped with a pentafluorophenyl moiety; compare data of **2** and **7** in Table 3 and literature values of **10** (Scheme 4), which are very similar to those of **7**.^43^ For **5**, which does not possess the 1,7-alkyl substituents, the dihedral angle between the BODIPY core and the phenyl moiety amounts to only approximately 60° (see above), indicating a partial conjugation of *meso*-substituent and BODIPY core and a higher rotational freedom. The first is supported by the bathochromic shift of the spectra compared with **6** and the latter by the lower fluorescence quantum yield of<0.2, which is consistent with the higher *k_nr_* value. Distinct bathochromic shifts were found for the absorption and emission maxima in toluene for **1**, **2** and **5**–**7**, which are caused by dispersive interactions between solvent and solute; toluene has a refractive index (*n*_D_) close to 1.5, whereas all other solvents employed possess values of 1.3<*n*_D_<1.4.

Elongation of the π system by attachment of styryl substituents at the 3,5-positions distinctly affects the spectral characteristics of the dyes by shifting the absorption and emission maxima to longer wavelengths (Figure 5). In the case of **8**, the shift amounts to +110 nm relative to **7**.^62^, ^70^–^72^ In the case of **3** and **4**, however, the shift is even larger relative to **2** with values of +140 and +200 nm, respectively, depending on solvent polarity (Table 3). Similar effects have been observed previously for doubly (*N*,*N*-dimethylamino)styryl-substituted BODIPYs.^62^ Moreover, not only are the band positions solvatochromically shifted but the fluorescence quantum yields are affected as well, resulting in a gradual decrease of the *Φ*_f_ value as the polarity of the solvent increases. Apparently, the charge-transfer character from the terminal electron-donating dimethylamino groups to the more electron-deficient BODIPY core is enhanced in polar solvents. Although one would perhaps expect a stronger displacement to the NIR for **4** compared with **3**, because the naphthenyl moieties should provide enhanced π-electron delocalization, the opposite effect is observed. The latter is tentatively ascribed to the higher steric demand of the naphthenyl moieties, which decreases the degree of planarity within the two naphthostyryl extensions. Although, unfortunately, all attempts to grow crystals of **4** suitable for X-ray structural analysis failed, quantum chemical calculations provided further insight into the molecular peculiarities of **3** and **4**. Whereas the dihedral angles between the BODIPY plane and the *meso*-substituent differ by only 0.4° and the angles between the two pyrrole units of the BODIPY core by only 0.8°, the average angle between the styryl extensions and the BODIPY core is much higher in modelled **4**, **4**^mod^, (41.8°) compared with **3**^mod^ (23.6°), as is the angle between the two styryl extensions (cf. 33.0° for **4** ^mod^ and 20.2° for **3**^mod^; see Table S3). Another noteworthy difference are the significantly larger Stokes shifts of **4** compared with **3**, which is likely to arise from an in-plane rotation of the naphthenyl moieties in the excited state, strengthening π conjugation and therefore lowering the energy of the excited state (Figure 5). In the case of **8**, for which the X-ray data revealed a more asymmetric nature and a significant flexibility of the CF_3_ groups even in the crystalline state, the much broader absorption band (Figure 5), which is also distinctly broader than that of other distyryl-extended BODIPYs,^70^ suggests that this behaviour is preserved in solution. Only upon excitation, charge redistribution apparently leads to a planarization and conformational freezing of the dye so that a typical narrow emission band is found (Figure 5). In addition, the *Φ*_f_ values of **8** are characteristically high (Table 3).

As the fluorine substitution at the *meso*-phenyl group is intended to equip the dyes with an increased photostability, **2**, **3**, **5**, **7** and **8** were compared to **10** under intense laser irradiation conditions (see Supporting Information for details). In fact, all the fluorinated dyes show a significantly higher photostability under intense illumination, with **8**, **3** and especially **5** being the most photostable. Interestingly, of the dipyrrin dyes, only the photostability of **5** is comparable to those of the styryl-extended dyes **3**, **4** and **8**, which show virtually none or only minor photobleaching even under intense long-term irradiation, as shown in the Supporting Information and previously by us.^46^–^48^

### Labelling of surface functional groups

**Figure 6:**
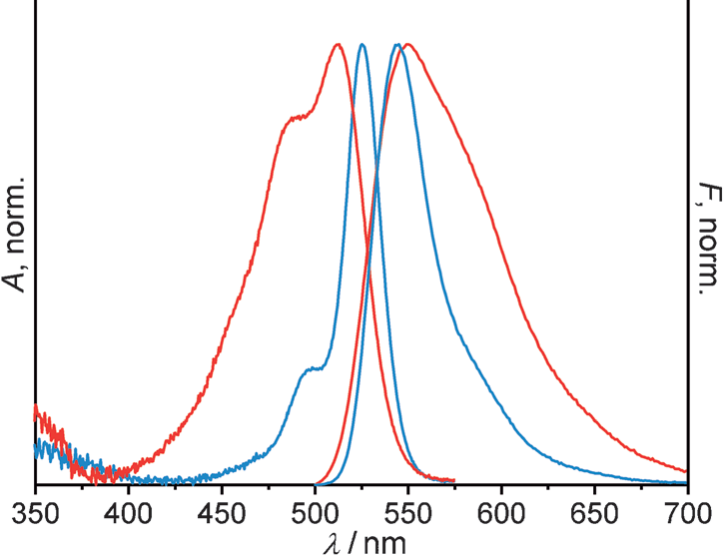
Absorption and emission spectra of **5** (**—**) and the reaction product of **5** and APTES (monosubstitution, **—**) in toluene, *λ*_exc_=490 nm.

Having a selection of pentafluorophenyl-substituted BODIPY dyes in hand, we strived to follow the work reported in Ref. 52 and tried to couple **1** and **2** to amino-functionalized glass slides. However, we were not able to observe significant binding to the amino surfaces, that is, we could only detect rather low fluorescence intensities after coupling of these compounds. Apparently, despite testing various modifications of the original protocol,^52^ the susceptibility of the pentafluorophenyl moiety for nucleophilic attack is not high enough to allow a high-yield reaction with surface-bound amino groups. We thus developed an alternative coupling protocol relying on an active chloro group in the 3-/5-position of the BODIPY core and utilized **5** for the purpose. BODIPY **5** combines five main advantages over previously reported substances for a quantitative and sensitive surface group analysis, that is, two amino-reactive chlorine moieties at the 3- and 5-position of the dipyrrin core that can undergo a monosubstitution reaction with nucleophiles under mild reaction conditions, a suitable amount of fluorine atoms (21.6 at- %) for quantitative assessment by XPS, a reasonable brightness for rapid analysis through fluorescence scanning, an absorption band well within the excitation range of commercial fluorescence scanners, and a change in its absorption and fluorescence maxima as a consequence of exchanging a chloro group by an amino group after a successful labelling reaction.^57^ As shown in Figure 6, reaction of **5** with a primary alkylamine results in a significant hypsochromic shift of the absorption band and a bathochromic shift and broadening of the emission band. Moreover, the reaction conditions can be adjusted in such a manner that either mono- or disubstitution occurs.^73^, ^74^ In addition, the mono- and disubstituted products can be distinguished by spectroscopic means, and both possess spectroscopic features still different from those of the unreacted dichloro derivative,^73^, ^74^ allowing for facile control at any stage of the reaction. Our control studies in solution have shown that at room temperature only one chlorine atom is substituted whereas at 110 °C, the substitution of both chlorine functionalities can be accomplished.

Up to now, to the best of our knowledge, probe molecules that can be used for the labelling of surface functional groups and can directly be analysed by an optical scanning and an elemental analysis technique such as XPS have not been reported. The common scenario either requires the removal of the label from various supports prepared for XPS analysis and its separate quantification by an optical method or the labelling of two different substrates at different concentrations, one suitable for XPS and the other for fluorescence analysis; in other cases, the dyes have to be excited by irradiation in the UV and escape fluorescence scanner excitation.^75^ Besides the preparation and characterization of the title compounds, the main aim of this work thus was to show the general suitability of our newly developed dyes for dual analysis of individual labelled supports by fluorescence and XPS. For this purpose, amino slides were prepared from cleaned and activated commercial glass slides using a vapour deposition procedure adapted from Ref. 76. The amount of 3-aminopropyltriethoxysilane (APTES) used in the functionalization procedures was chosen in such a way that a quantitative surface functionalization is reached. After washing and drying, the functionalized slides were incubated for 24 h with a 0.2 mm solution of **5** in acetonitrile under stirring at room temperature. A main question here was whether for surfaces covered densely with amino groups, the amount of dye conjugated to the support is appropriate for unequivocal fluorine atom detection by XPS while at the same time fluorescence scanning does not suffer from signal saturation problems.

### Fluorescence analysis of slides

**Figure 7:**
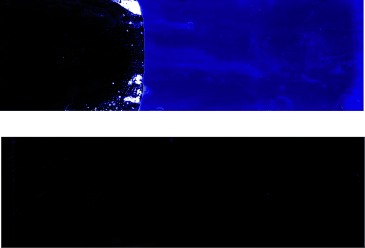
Scan images of amino-functionalized slide prior to (bottom) and after reaction with **5** (top; the part of the slide shown on the left was not functionalized to facilitate handling) as described in the text; *λ*_exc_=488 nm, *λ*_em_=550–600 nm (standard green filter in emission), photomultiplier tube (PMT) voltage=500 V.

Figure 7 shows two representative images obtained with a fluorescence scanner operating at 488 nm excitation of amino-functionalized slides, one reacted in the above-mentioned way with **5** and the other one in its native state. Table 4 collects the relevant fluorescence data retrieved from the scans. Given a working range of 300–800 V of the scanner’s photomultiplier tube, the parameter used for the present measurements (500 V; see Figure 7) makes it obvious that not only the labelling procedure yields rather uniform surface coverage but that the approach realized here leaves enough room for higher and lower signal intensities, that is, changes in dye concentration.

**Table 4:** Fluorescence scanner data of investigated slides

*F*_*λ*exc_[a]	bare glass[b]	APTES[c]	APTES+5[I][d]	APTES+5[II][d]	5[e]
	A[f]	B[f]	C[f]	D[f]	E[f]
av_488_	17	35	23 255	12 740	194
SD_488_	±17	±23	±3803	±935	±82
av_532_	12	11	7416	5395	72
SD_532_	±10	±12	±991	±268	±38

[a] Average (av) arbitrary fluorescence intensity per pixel and standard deviation (SD) for excitation at 488 and 532 nm.

[b] Piranha-cleaned slide.

[c] APTES-functionalized slide.

[d] Two different APTES-functionalized slides reacted with **5**.

[e] Piranha-cleaned slide (without APTES), treated with **5** and subjected to normal washing/drying procedure.

[f] Slide abbreviation.

### X-ray photoelectron spectroscopy

**Scheme 5:**
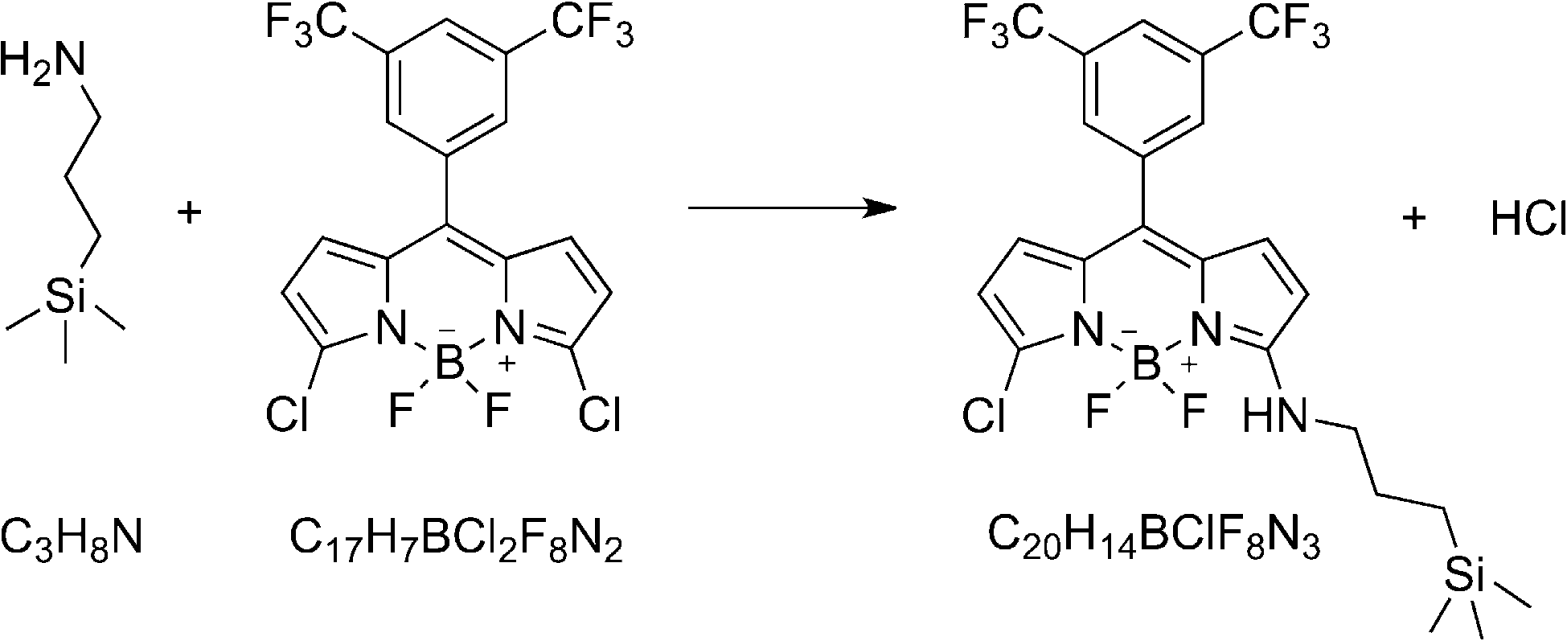
Labelling reaction.

XPS analyses were employed on the same slides measured previously in the scanner to quantitatively evaluate the different derivatization steps. Through the assessment of atomic ratios and atomic concentrations (at- %) by this elemental analysis technique, it is possible to determine for instance the entire nitrogen or fluorine content introduced by functionalization with APTES and subsequent reaction with a dye such as **5**. On the basis of the labelling reaction depicted in Scheme 5, the amount fraction of carbon atoms containing amino groups [NH_2_]_C_ that are reactive towards **5** can be derived from the fluorine content according to Equation (1).^13^, ^17^, ^77^–^81^


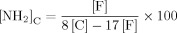
(1)

**Figure 8:**
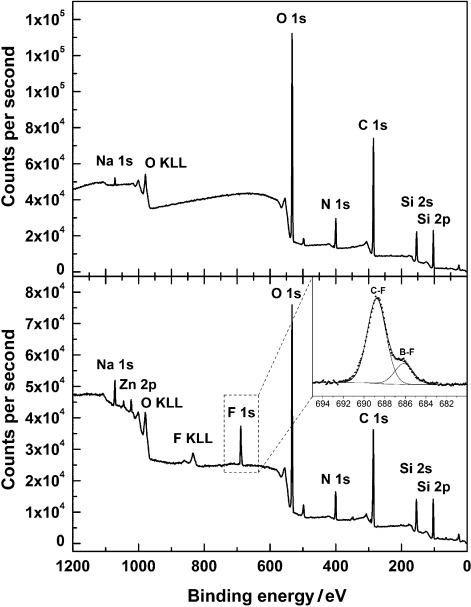
XPS survey scan spectra of the dye-covered area of the slides shown in Figure 7, amino-functionalized slide prior to (slide B, top) and after reaction with **5** (slide D, bottom; inset shows high-resolution F 1s core level spectra) as described in the text.

Equation (1) follows from the generalized mass balance law −(CH_2_)_3_–(NH_2_)_1_ +*y* (CF_3_)_2_C_6_H_3_(C_9_H_4_BF_2_N_2_)Cl_2_→C_3+17*y*_N_1+2*y*_F_8*y*_+HCl of the reaction under consideration of *y*=[F]/8, *x*=[C]−17*y*, and Equation (2). Table 5 lists representative XPS results, and Figure 8 shows the corresponding XPS scans.


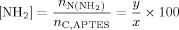
(2)

**Table 5:** Formal XPS elemental composition [at- %] of investigated slides and relevant atomic number ratios

EC[a]	bare glass[b]	APTES[c]	APTES+5(I)[d]	APTES+5(II)[d]	APTES+5(II)[e]	5[f]
	A[g]	B[g]	C[g]	D^0[g]^	D^60[g]^	E[g]
C	6.9	51.3	42.7	44.4	51.4	9.2
N	0.6	7.4	7.1	7.2	8.4	1.1
O	69.1	28.1	30.4	28.3	21.4	67.0
Si	23.4	13.2	16.7	15.3	10.6	22.7
F	–	–	3.0	4.8	8.1	–
N/C	0.09	0.14	0.17	0.16	0.16	0.12
C/Si	0.30	3.89	2.56	2.90	4.85	0.41
N/Si	0.03	0.56	0.42	0.47	0.79	0.05

[a] EC=elemental composition and atom number ratios; elemental composition was recalculated for the relevant elements, see Table S4 for further details.

[b] Piranha-cleaned slide.

[c] APTES-functionalized slide.

[d] Two different APTES-functionalized slides reacted with **5**.

[e] Slide D observed at an electron emission angle of 60° instead of 0°; all other measurements were performed at 0°.

[f] Piranha-cleaned slide (without APTES), treated with **5** and subjected to normal washing/drying procedure.

[g] Slide abbreviation.

As a reference, we also analysed bare slides only activated with Piranha solution (slide A, Table 5) as well as Piranha-treated slides not further functionalized but only subjected to the labelling procedure (slide E). The latter thus provides a measure for the amount of unspecific binding of **5** to a bare support and for the quality of the washing/drying process. The XPS results of the Piranhaactivated (slide A) and the dye-treated, washed substrates (slide E) revealed high amounts of silicon and oxygen that are specific for the glass substrate, as well as other glass ingredients (Ca, Zn, Mg, Al) and small amounts of organic (hydrocarbon) contaminations (originating from incomplete substrate cleaning and/or adsorbed impurities from activation or washing procedures). In line with only slightly enhanced fluorescence from slide E compared with slide A (Table 4), which might either be due to traces of adsorbed **5** or to slightly enhanced scattering because of the deposition of unspecific organic material on the support during the processing, these results suggest that unspecific adsorption of **5** is negligible.

The content of the carbon and nitrogen components increased significantly after functionalization with APTES to generate amino-terminated surfaces (slide B). High-resolution spectra of the N 1s region exhibit two components at 399.4 and 401.1 eV, corresponding to amino groups (NH_2_) and their tightly electrostatically bound ammonium counterparts (NH_3_^+^⋅⋅⋅X^−^), which lie at energies between the more common hydrogen-bonded and protonated amino groups (Figure S2).^78^, ^82^ After the labelling process, a new fluorine peak with two components at 688.5 eV and 686.2 eV related to the CF_3_ and BF_2_ moieties of bound dye is detected in the survey and high-resolution XPS spectra for slides D and E (Figure 8, Figure S3). The presence of fluorine groups can be verified independently by a new CF_3_ component at 293.3 eV in the high-resolution C 1s core level spectra after treatment of the APTES-functionalized slide with **5** (Figure S4). These fluorine-related signals were absent on the reference supports (slides A and E). In addition, traces of about 0.2 at- % of chlorine could be detected for the APTES+**5** slides D, supporting the successful labelling step. Along with the fact that the **5**-tethered slides show spectral features of the monosubstituted reaction product between **5** and APTES (cf. absorption spectra in Figure 6 and fluorescence excitation spectra in Figure S5), these chlorine traces by XPS support the presence of only the monosubstitution product on the slide. The absence of the fluorine peak in the case of slide E suggests that an activated glass slide without amino moieties is unable to react with **5** when incubated for 24 h at room temperature, despite the fact that nucleophilic hydroxyl groups are present on the surface of the plain activated SiO_2_ supports, which is again supported by the respective fluorescence scanning results of slide E in Table 4. Moreover, the data listed for slide D at 60° electron emission angle in Table 5 (slide D^60^) further show that the amount of fluorine atoms attached to the outermost layer of the APTES film increases significantly to about 8 at- % when measured in a more surface-sensitive mode to suppress substrate signals (Si and O). If we assume that the amino group density achieved under the high-loading deposition conditions as employed here amounts to≥3×10^14^ molecules per cm^2^,^76^, ^83^ the concentration of labelled amino groups derived from slide D^60^ data of [NH_2_]_C_∼3 % according to Equations (1) and (2) indicates that about 9×10^12^ NH_2_ groups per cm^2^ have been successfully labelled with **5**. Considering that based on the van der Waals radii of common organic dye molecules such as rhodamines or BODIPYs the theoretically achievable surface coverage amounts to 2.5×10^13^ per cm^2^,^25^ the present labelling ratio of only approximately 3 % suggests that the high-loading deposition procedure adapted from Ref. 76 indeed produces APTES films, which are thicker than a monolayer. Such a behaviour has been reported before for APTES and other deposition techniques.^84^, ^85^ Apparently, the opposed trends found in the XPS and fluorescence results discussed above, that is, a decrease in fluorescence intensity upon going from slide C to D yet a slight increase in fluorine content, indicates that more dye molecules undergo unspecific interaction with the thicker APTES film on slide D or interact with each other, leading to self-quenching effects. This is supported by the differences in fluorescence signals obtained from 488 and 532 nm excitation (Table 4) as well as by the red-shifted fluorescence spectra of slide D (Figure S5), longer wavelengths usually leading to preferred excitation of dye species that are energetically stabilized through interaction with the surface or other dye molecules.^86^

## Conclusion

The work presented here describes the preparation, crystallographic and spectroscopic characterization of a family of boron-dipyrromethene (BODIPY) dyes containing various fluorinated substituents. Whereas the spectral features of the dyes are typical for such type of BODIPYs, some of the dyes show an exceptional brightness, especially **1** and **2**, and virtually all of them an improved photostability. Interestingly, control of the conformation and planarity of the styryl extensions in red/NIR-emitting BODIPYs **3** and **4** by choice of the styryl’s aromatic unit allows a tailoring of the Stokes shifts of such dyes to a significant extent. Deviations from planarity and symmetric arrangement in the ground state and a planarization upon excitation also govern the spectroscopic behaviour of nondonor styryl-extended BODIPYs such as **8**.

A particularly exciting aspect of our work is the successful use of the very photostable compound **5** as a dual XPS/fluorescence label, which allows to obtain quantitative information on the chemical composition from selected spots of a labelled substrate after exactly the same substrate has been screened by fluorescence scanning without the need of any additional processing step. As we have shown, highly 3-aminopropyltriethoxysilane (APTES)-covered surfaces are detectable by opposite trends in the XPS and fluorescence data, presumably due to the stronger interaction of dye molecules attached to multi-layer type, irregular APTES films with a higher surface roughness. The fluorine contents found for the labelled slides prepared here suggest that also the less densely APTES-functionalized slides can be analysed by this method combination. However, if the functional group density on the surface would be reduced by two orders of magnitude or more, a next generation of fluorescent labels with an even higher fluorine content has to be developed. Currently such work is in progress in our laboratories.

## Experimental Section

### General

All reagents were obtained from commercial suppliers and used without further purification unless otherwise indicated. All air- and moisture-sensitive reactions were carried out under argon atmosphere in oven-dried glassware. TLC was performed on Merck silica gel 60 F254 TLC plates with a fluorescent indicator for 254 nm excitation. Compounds were visualized under UV light at 254 nm. Column chromatography was carried out with Merck silica gel 60 (0.040–0.063 mm) using the eluents specified (Hex=hexane, PE=petroleum ether). NMR measurements were carried out on a Bruker AV 400 or on a Bruker AVANCE III 500 at 27 °C using residual protonated solvent signals as internal standard (^1^H: δ [CDCl_3_]=7.26 ppm and ^13^C: δ [CDCl_3_]=77.16 ppm). Assignments are based on chemical shifts and/or DEPT spectra (Ar is used as abbreviation for assigning aromatic moieties). ^19^F NMR assignments are based on multiplicities and integration of correlated signals. HRMS was performed with a Thermo Scientific Exactive Orbitrap in the positive ion mode using the Thermo Xcalibur operating and data acquisition software or on a Waters LCT Premier XE. UPLC was performed with a Waters UPLC Acquity equipped with a Waters LCT Premier XE mass detector for UPLC-HRMS, with Waters Alliance systems (consisting of a Waters Separations Module 2695, a Waters Diode Array detector 996 and a Waters Mass Detector ZQ 2000) equipped with a Acquity BEH C18 (2.1×50 mm) column. The melting points (mp) measured with a hot stage microscope Galen III (Leica) are uncorrected. UV-vis absorption spectra were recorded on an Analytik Jena Specord 210 Plus spectrophotometer. Steady-state fluorescence measurements were carried out on a Horiba Jobin–Yvon FluoroMax-4P spectrofluorometer and a Spectronics Instrument 8100 spectrofluorometer, using standard 10 mm path length quartz cuvettes. Fluorescence lifetimes were determined with a unique customized laser impulse fluorometer with picosecond time resolution described elsewhere.^61^, ^87^ The fluorescence lifetime profiles were analysed using the Horiba Scientific software package DAS 6. All solvents employed for the spectroscopic measurements were of UV spectroscopic grade (Aldrich). Corning plain, pre-cleaned micro slides (75×25 mm) were used for further functionalization steps.

### X-ray structure analysis, X-ray diffraction

Single-crystal X-ray intensity data were collected at 216 K on a Bruker APEX-II diffractometer with a CCD-area detector, equipped with a graphite monochromator using Mo-Kα-radiation (*λ*=0.71073 Å). Data reductions and absorption corrections were performed with the Bruker AXS SAINT^88^, ^89^ and SADABS^90^ packages, respectively. The structures were solved by direct methods and refined by full-matrix least-squares calculations using the programs SHELXS/L^91^, ^92^ Anisotropic displacement parameters were employed for nonhydrogen atoms. The hydrogen atoms were treated isotropically with *U*_iso_=1.2 times the *U*_eq_ value of the parent atom. CCDC-893399–893403 and 905368 contain the supplementary crystallographic data (excluding structure factors) for this paper. These data can be obtained free of charge from The Cambridge Crystallographic Data Centre via http://www.ccdc.cam.ac.uk.

### Scanning and data analysis

All glass slides were scanned at 488 nm and 532 nm using a Molecular Devices Axon GenePix® 4300 A fluorescence scanner at 20 μm resolution. These signals were collected with 16-bits-per-pixel resolution. The images were analysed with the GenePix Pro 7.1 software provided by the manufacturer. A grid of 5×6 spots (each 3 mm in diameter) was used to average the fluorescence signal on the dye covered area, and the background was averaged using a grid of the same dimensions on the uncovered area.

### X-ray photoelectron spectroscopy

XPS measurements were carried out with an Kratos Analytical AXIS Ultra DLD photoelectron spectrometer. XPS spectra were recorded using monochromated Al Kα excitation at pass energies of 80 eV for survey and 20 eV for high-resolution core-level spectra. Additionally, the charge neutralization system was applied. The electron emission angle was 0° or 60° and the source-to-analyzer angle was 60°. The binding energy scale of the instrument was calibrated following a Kratos Analytical procedure, which uses ISO 15472 binding energy (BE) data.^93^, ^94^ The binding energy scale was corrected for static charging by using BE=285.0 eV for the aliphatic C 1s component.^95^ High resolution C 1s, N 1s, and F 1s core level spectra were analysed using the Casa Software CasaXPS peak fit program. In curve fitting of core level spectra, full widths at half maximum were constrained to be equal and a Gaussian/Lorentzian product function peak shape model (G/L=30) was used in combination with a Shirley background.

### Synthesis

**General procedure for BODIPYs 1, 2, 6 and 7**: BODIPYs were prepared according to Ref. 54. The corresponding aldehyde (4.0 mmol, 1.0 equiv) and pyrrole (8.0 mmol, 2.0 equiv) were dissolved in dry CH_2_Cl_2_ (70 mL) under argon. A few drops of trifluoroacetic acid (TFA) were added and the solution was stirred at RT in the dark until total consumption of the aldehyde (monitored by TLC). Tetrachloro-*p*-benzoquinone (983.5 mg, 4.0 mmol, 1.0 equiv) was added and the mixture was stirred for an additional 5 min. The reaction mixture was then treated with *N*,*N*-diisopropylethylamine (DIPEA; 5.0 mL, 28.0 mmol, 7.0 equiv) and BF_3_**⋅**OEt_2_ (5.5 mL, 44.0 mmol, 11.0 equiv). After stirring for 15 min, the dark solution was washed with H_2_O (3×50 mL). After extraction of the aqueous phase with CH_2_Cl_2_ (3×50 mL), the combined organic solutions were dried over Na_2_SO_4_, filtered and concentrated in vacuo. The crude product was purified by silica-gel flash column chromatography (CH_2_Cl_2_/PE or toluene as eluent).

**8-(2,3,4,5,6-Pentafluorophenyl)-1,3,5,7-tetramethyl-4,4-difluoro-4-bora-3 a,4 a-diaza-*s*-indacene (1)**: Compound **1** was obtained as orange crystals (414 mg, 25 %): mp: 231 °C; ^1^H NMR (500 MHz, CDCl_3_): *δ*=1.62 (s, 6 H, 2×C*H*_3_), 2.57 (s, 6 H, 2×C*H*_3_), 6.06 ppm (s, 2 H, 2×C*H*); ^19^F NMR (471 MHz, CDCl_3_): *δ*=−159.77 (dt, 2 F, *J=*21.3, 7.0 Hz; 2×F_ar_), −150.77 (t, 1 F, *J=*20.9 Hz, F_ar_), −146.39 (dd, 2 F, *J*=64.5, 32.1 Hz, BF_2_), −139.50 ppm (dd, 2 F, *J=*21.9, 7.3 Hz, 2×F_ar_); MS (ESI+): *m*/*z* [*M*+H]^+^ calcd for C_19_H_14_BF_7_N_2_: 415.122, found: 415.128.

**8-(2,3,4,5,6-Pentafluorophenyl)-1,3,5,7-tetramethyl-2,6-diethyl-4,4-difluoro-4-bora-3 a,4 a-diaza-*s*-indacene (2)**: Compound **2** was obtained as orange-red crystals (1.04 g, 55 %): mp: 206 °C; ^1^H NMR (400 MHz, CDCl_3_): *δ*=1.02 (t, 6 H, *J*=7.6 Hz, 2×C*H*_3_), 1.51 (s, 6 H, 2×C*H*_3_), 2.34 (q, 4 H, *J*=7.6 Hz, 2×C*H*_2_–CH_3_), 2.55 ppm (s, 6 H, 2×CH_2_–C*H*_3_); ^19^F NMR (471 MHz, CDCl_3_): *δ*=−160.07 (dt, 2 F, *J*=22.8, 7.9 Hz, 2×F_ar_), −151.30 (t, 1 F, *J*=20.6 Hz, F_ar_), −145.96 (dd, 2 F, *J*=65.4, 32.2 Hz, BF_2_), −139.47 ppm (dd, 2 F, *J*=22.7, 7.0 Hz, 2×F_ar_); HRMS (ESI+): *m*/*z* [*M*+H]^+^ calcd for C_23_H_22_BF_7_N_2_: 471.1837, found: 471.1836.

**8-(3,5-Bis(trifluoromethyl)phenyl)-1,3,5,7-tetramethyl-4,4-difluoro-4-bora-3 a,4 a-diaza-*s*-indacene (6)**: Compound **6** was obtained as orange crystals (350 mg, 19 %): mp: 180 °C; ^1^H NMR (500 MHz, CDCl_3_): *δ*=1.36 (s, 6 H, 2×C*H*_3_), 2.60 (s, 6 H, 2×C*H*_3_), 6.07 (s, 2 H, 2×C*H*), 7.84 (s, 2 H, 2×C*H*_ar_), 8.04 ppm (s, 1 H, C*H*_ar_); HRMS (ESI+): *m*/*z* [*M*+H]^+^ calcd for C_21_H_17_BF_8_N_2_: 461.1429, found: 461.1422.

**8-(3,5-Bis(trifluoromethyl)phenyl)-1,3,5,7-tetramethyl-2,6-diethyl-4,4-difluoro-4-bora-3 a,4 a-diaza-*s*-indacene (7)**: Compound **7** was obtained as a greenish red, shining solid (929 mg, 45 %): mp: 175 °C; ^1^H NMR (500 MHz, CDCl_3_): *δ*=0.99 (t, 6 H, *J=*7.6 Hz, 2×CH_2_–C*H*_3_), 1.22 (s, 6 H, 2×C*H*_3_), 2.31 (q, 4 H, *J*=7.6 Hz, 2×C*H*_2_–CH_3_), 2.54 (s, 6 H, 2×C*H*_3_), 7.84 (s, 2 H, 2×C*H*_ar_), 8.03 ppm (s, 1 H, C*H*_ar_); HRMS (ESI+): *m*/*z* [*M*+H]^+^ calcd for C_25_H_25_BF_8_N_2_: 517.2055, found: 517.2046.

**8-(3,5-Bis(trifluoromethyl)phenyl)-3,5-dichloro-4,4-difluoro-4-bora-3 a,4 a-diaza-*s*-indacene (5)**: Pyrrole (50.0 mL, 720 mmol, 25.0 equiv) and 3,5-bis(trifluoromethyl)benzaldehyde (6.97 g, 28.8 mmol, 1.0 equiv) were added to a dry round-bottomed flask and degassed with argon for 10 min. TFA (222 μL, 2.88 mmol, 0.10 equiv) was added, and the solution was stirred under argon at RT for 30 min. After quenching with NaOH (50 mL, 0.1 m), EtOAc (50 mL) was added. The organic phase was washed with H_2_O, dried over Na_2_SO_4_, and after extraction with EtOAc (50 mL), the solvent was removed in vacuo to afford a brown oil. Excess pyrrole was removed trough bulb-to-bulb distillation (80 °C, 0.1×10^−3^ bar). After purification by silica-gel flash column chromatography (1:1 *v*/*v* CH_2_Cl_2_/PE), the brown solid (8.0 g) was dissolved in dry tetrahydrofuran (THF; 300 mL). The solution was purged with argon and cooled to −78 °C, and a suspension of *N*-chlorosuccinimide (8.20 g, 61.4 mmol, 2.0 equiv) in THF (50 mL) was added. The reaction mixture was stirred at −78 °C for 2 h and placed in the freezer overnight at −20 °C. After stirring at RT for an additional 5 h, H_2_O (150 mL) was added. Following extraction with CH_2_Cl_2_, the organic layer was dried over Na_2_SO_4_ and filtered, and the solvent was removed under reduced pressure, and the residue was chromatographed on silica gel (1:2 *v*/*v* CH_2_Cl_2_/PE). The intermediate (4.7 g, 0.011 mol, 1.0 equiv) was dissolved in CH_2_Cl_2_ (200 mL), and 2,3-dichloro-5,6-dicyano-1,4-benzoquinone (DDQ; 2.5 g, 0.011 mol, 1.0 equiv) in CH_2_Cl_2_ (70 mL) was added. After stirring for 1 h at RT, *N*,*N*,*N*-triethylamine (TEA; 20 mL, 0.144 mmol, 13.0 equiv) and BF_3_**⋅**OEt_2_ (20 mL, 0.160 mmol, 20.0 equiv) were added slowly under vigorous stirring. After stirring overnight, the solvent was removed in vacuo. The dark brown residue was dissolved in CH_2_Cl_2_ (100 mL) and washed with NaHCO_3_ (5 % *m*/*v*, 1×80 mL) and H_2_O (1×80 mL). After extraction of the aqueous phase with CH_2_Cl_2_ (3×100 mL), the combined organic solutions were dried over Na_2_SO_4_, filtered and concentrated in vacuo. The crude product was purified by silica-gel flash column chromatography (4:6 *v*/*v* CH_2_Cl_2_/PE). Red crystals of **5** were obtained (882 mg, over all 6 %): mp: 228 °C; ^1^H NMR (500 MHz, CDCl_3_): *δ*=6.51 (d, 2 H, *J*=4.4 Hz, 2×C*H*), 6.72 (d, 2 H, *J*=4.4 Hz, 2×C*H*), 7.98 (s, 2 H, 2×C*H*_ar_), 8.11 ppm (s, 1 H, 2×C*H*_ar_); HRMS (ESI−): *m*/*z* [*M*−F]^−^ calcd for C_17_H_7_BF_8_N_2_Cl_2_: 452.9962, found: 452.9979.

**BODIPYs 3 and 4**: **3** and **4** were prepared as described earlier.^46^–^48^

**Bis-(3,5-(3,5-bis(trifluoromethyl)phenyl)vinyl)-8-(3,5-bis(trifluoromethyl)phenyl)-1,7-dimethyl-2,6-diethyl-4,4-difluoro-4-bora-3a,4a-diaza-*s*-indacene (8)**: Compound **7** (0.103 g, 0.2 mmol, 1.0 equiv) and 3,5-bis(trifluoromethyl)benzaldehyde (74 μL, 0.46 mmol, 2.3 equiv) were dissolved in CH_2_Cl_2_ (10 mL) and dimethylformamide (DMF; 10 mL ). After addition of glacial acetic acid (150 μL, 2.6 mmol, 13.0 equiv) and piperidine (180 μL, 3.1 mmol, 15.5 equiv), the reaction mixture was refluxed for 26 h with a small amount of 4 Å molecular sieves. The solvents were evaporated in vacuo, and the blue solid was directly chromatographed on silica (3:7 *v*/*v* CH_2_Cl_2_/PE) to obtain cyan needles of **8** (41.0 mg, 21 %): mp: 303 °C; ^1^H NMR (500 MHz, CDCl_3_): *δ*=1.21 (t, 6 H, *J=*7.6 Hz, 2×CH_2_–C*H*_3_), 1.31 (s, 6 H, 2×C*H*_3_), 2.64 (q, 4 H, *J=*7.5 Hz, 2×C*H*_2_–CH_3_), 7.35 (d, 2 H, *J=*16.8 Hz, CH=CH), 7.84 (s, 2 H, 2×C*H*_ar_), 7.85 (d, 2 H, *J=*16.7 Hz, CH=CH), 7.91 (s, 2 H, 2×C*H*_*a*r_), 7.99 (s, 4 H, 4×C*H*_ar_), 8.10 ppm (s, 1 H, C*H*_ar_); MS (ESI−): *m*/*z* [*M*−H]^−^ calcd for C_43_H_29_BF_20_N_2_: 963.203, found: 963.200.

**Activation and functionalization of glass slides**: Commercial glass slides were activated with freshly prepared piranha solution (3:1 *v*/*v* concd H_2_SO_4_/H_2_O_2_) for 2 h, washed thoroughly with MilliQ H_2_O and stored under H_2_O, no longer than one week prior to use. According to a recent publication, the best aminosilane functionalization is achieved by using a vapour deposition procedure.^76^ For this purpose, 3-aminopropyltriethoxysilane (APTES; 100 μL, 10 % *v*/*v*) in dry toluene were added into a 125 mL PFA bottle under argon, and two activated and carefully dried glass slides were placed in the bottle, which was heated for 2 h at 150 °C. The slides were removed from the bottle and rinsed with acetone and *iso*-propanol, followed by sonication in *iso*-propanol, rinsing with *iso*-propanol and H_2_O, and drying with a slide centrifuge. The washed and dried slides were placed in a solution of **5** in MeCN (25 mL, 0.2 mm) in a 50 mL centrifuge tube and incubated overnight under stirring at RT. The slides were subsequently washed as described for the functionalization (see above). The functionalized and incubated slides were stored under argon and removed from the protecting atmosphere just prior to the measurements.

### Computational details

The optimization of the S_0_ ground state geometries in the gas phase was performed with the density functional theory (DFT) method employing the hybrid functional B3LYP with a 6–31G basis set and energy-minimized as implemented in Gaussian 03.^96^

## Supplementary material

As a service to our authors and readers, this journal provides supporting information supplied by the authors. Such materials are peer reviewed and may be re-organized for online delivery, but are not copy-edited or typeset. Technical support issues arising from supporting information (other than missing files) should be addressed to the authors.

## References

[1] Beier M, Hoheisel JD (1999). Nucleic Acids Res.

[2] del Campo A, Bruce I (2005). Top. Curr. Chem.

[3] Jonkheijm P, Weinrich D, Schröder H, Niemeyer CM, Waldmann H (2008). Angew. Chem.

[b97] (2008). Angew. Chem. Int. Ed.

[4] Park S, Lee M-R, Shin I (2009). Bioconjugate Chem.

[5] Queffélec C, Petit M, Janvier P, Knight DA, Bujoli B (2012). Chem. Rev.

[6] Rożkiewicz DI, Ravoo BJ, Reinhoudt DN, Rurack K, Martínez-Máñez R (2010). The Supramolecular Chemistry of Organic-Inorganic Hybrid Materials.

[7] Holländer A, Kröpke S, Pippig F (2008). Surf. Interface Anal.

[8] Abbas A, Vivien C, Bocquet B, Guillochon D, Supiot P (2009). Plasma Processes Polym.

[9] Kim J, Shon HK, Jung D, Moon DW, Han SY, Lee TG (2005). Anal. Chem.

[10] Oran U, Swaraj S, Lippitz A, Unger WES (2006). Plasma Processes Polym.

[11] Truica-Marasescu F, Girard-Lauriault P-L, Lippitz A, Unger WES, Wertheimer MR (2008). Thin Solid Films.

[12] Girard-Lauriault P-L, Desjardins P, Unger WES, Lippitz A, Wertheimer MR (2008). Plasma Processes Polym.

[13] Yegen E, Lippitz A, Treu D, Unger WES (2008). Surf. Interface Anal.

[14] Boulares-Pender A, Prager-Duschke A, Elsner C, Buchmeiser MR (2009). J. Appl. Polym. Sci.

[15] Fally F, Doneux C, Riga J, Verbist JJ (1995). J. Appl. Polym. Sci.

[16] Keen I, Broota P, Rintoul L, Fredericks P, Trau M, Grøndahl L (2006). Biomacromolecules.

[17] Graf N, Lippitz A, Gross T, Pippig F, Holländer A, Unger WES (2010). Anal. Bioanal. Chem.

[18] Holländer A, Pippig F, Dubreuil M, Vangeneugden D (2008). Plasma Processes Polym.

[19] Pippig F, Sarghini S, Holländer A, Paulussen S, Terryn H (2009). Surf. Interface Anal.

[20] Briggs D, Seah MP (1990). Practical Surface Analysis: Auger and X-ray Photoelectron Spectroscopy.

[21] Ghasemi M, Minier M, Tatoulian M, Arefi-Khonsari F (2007). Langmuir.

[22] Coussot G, Perrin C, Moreau T, Dobrijevic M, Le Postollec A, Vandenabeele-Trambouze O (2011). Anal. Bioanal. Chem.

[23] Noel S, Liberelle B, Robitaille L, De Crescenzo G (2011). Bioconjugate Chem.

[24] Holländer A (2004). Surf. Interface Anal.

[25] Xing Y, Borguet E (2007). Langmuir.

[26] Shlyapnikova EA, Shlyapnikov YM, Afanas’ev VN, Afanas’eva GV, Gavryushkin AV, Beletskii IP (2007). Russ. J. Bioorg. Chem.

[27] Hoffmann K, Mix R, Resch-Genger U, Friedrich JF (2007). Langmuir.

[28] Pippig F, Holländer A (2007). Appl. Surf. Sci.

[29] Wilson R, Schiffrin DJ (1995). Analyst.

[30] Seah MP (1996). Philos. Trans. R. Soc. London Ser. A.

[31] Kim KJ, Unger WES, Kim JW, Moon DW, Gross T, Hodoroaba V-D, Schmidt D, Wirth T, Jordaan W, van Staden M, Prins S, Zhang L, Fujimoto T, Song XP, Wang H (2012). Surf. Interface Anal.

[32] Monte C, Resch-Genger U, Pfeifer D, Taubert DR, Hollandt J (2006). Metrologia.

[33] Ulrich G, Ziessel R, Harriman A (2008). Angew. Chem.

[b98] (2008). Angew. Chem. Int. Ed.

[34] Kobayashi H, Ogawa M, Alford R, Choyke PL, Urano Y (2010). Chem. Rev.

[35] Suzuki S, Kozaki M, Nozaki K, Okada K (2011). J. Photochem. Photobiol. C.

[36] Boens N, Leen V, Dehaen W (2012). Chem. Soc. Rev.

[37] Loudet A, Burgess K (2007). Chem. Rev.

[38] Ziessel R, Ulrich G, Harriman A (2007). New J. Chem.

[39] Treibs A, Kreuzer F-H (1968). Liebigs Ann. Chem.

[40] Rohand T, Qin W, Boens N, Dehaen W (2006). Eur. J. Org. Chem.

[41] Li L, Nguyen B, Burgess K (2008). Bioorg. Med. Chem. Lett.

[42] Rurack K, Kollmannsberger M, Resch-Genger U, Daub J (2000). J. Am. Chem. Soc.

[43] Trieflinger C, Röhr H, Rurack K, Daub J (2005). Angew. Chem.

[b99] (2005). Angew. Chem. Int. Ed.

[44] Wang Y-W, Descalzo AB, Shen Z, You X-Z, Rurack K (2010). Chem. Eur. J.

[45] Hecht M, Kraus W, Rurack K (2013). Analyst.

[46] Rurack K, Descalzo AB, Fischer T, Behnke T (2011). Difluoroboradiazaindacen-Farbstoffe.

[47] Rurack K, Descalzo AB, Fischer T, Behnke T (2011). Difluoroboradiazaindacen-Farbstoffe.

[48] Rurack K, Descalzo AB, Fischer T, Behnke T (2011). Difluoroboradiazaindacene Dyes.

[49] Funabiki K, Sugiyama N, Iida H, Jin J-Y, Yoshida T, Kato Y, Minoura H, Matsui M (2006). J. Fluorine Chem.

[50] Mitronova GY, Belov VN, Bossi ML, Wurm CA, Meyer L, Medda R, Moneron G, Bretschneider S, Eggeling C, Jakobs S, Hell SW (2010). Chem. Eur. J.

[51] Woydziak ZR, Fu L, Peterson BR (2012). J. Org. Chem.

[52] Vives G, Giansante C, Bofinger R, Raffy G, Guerzo AD, Kauffmann B, Batat P, Jonusauskas G, McClenaghan ND (2011). Chem. Commun.

[53] Alamiry MAH, Benniston AC, Hagon J, Winstanley TPL, Lemmetyinen H, Tkachenko NV (2012). RSC Adv.

[54] Galangau O, Dumas-Verdes C, Méallet-Renault R, Clavier G (2010). Org. Biomol. Chem.

[55] Littler BJ, Miller MA, Hung C, Wagner RW, O’Shea DF, Boyle PD, Lindsey JS (1999). J. Org. Chem.

[56] Baruah M, Qin W, Basarić N, De Borggraeve WM, Boens N (2005). J. Org. Chem.

[57] Rohand T, Baruah M, Qin W, Boens N, Dehaen W (2006). Chem. Commun.

[58] Picou CL, Stevens ED, Shah M, Boyer JH (1990). Acta Crystallogr. Sect. C.

[59] Wang D-C, He C, Fan J-L, Huang W-W, Peng X-J (2007). Acta Crystallogr. Sect. E.

[60] Zhou X (2010). Acta Crystallogr. Sect. E.

[61] Shen Z, Röhr H, Rurack K, Uno H, Spieles M, Schulz B, Reck G, Ono N (2004). Chem. Eur. J.

[62] Yu Y-H, Descalzo AB, Shen Z, Röhr H, Liu Q, Wang Y-W, Spieles M, Li Y-Z, Rurack K, You X-Z (2006). Chem. Asian J.

[63] Chen Y, Wan L, Zhang D, Bian Y, Jiang J (2011). Photochem. Photobiol. Sci.

[64] Mula S, Ray AK, Banerjee M, Chaudhuri T, Dasgupta K, Chattopadhyay S (2008). J. Org. Chem.

[65] Kollmannsberger M, Rurack K, Resch-Genger U, Daub J (1998). J. Phys. Chem. A.

[66] López Arbeloa F, Bañuelos Prieto J, Martínez Martínez V, Arbeloa López T, López Arbeloa I (2004). ChemPhysChem.

[67] Filarowski A, Kluba M, Cieślik-Boczula K, Koll A, Kochel A, Pandey L, De Borggraeve WM, Van der Auweraer M, Catalán J, Boens N (2010). Photochem. Photobiol. Sci.

[68] Rurack K, Saalfrank P, Daub J

[69] Swartz DL, Staples RJ, Odom AL (2011). Dalton Trans.

[70] Rurack K, Kollmannsberger M, Daub J (2001). New J. Chem.

[71] Qin W, Rohand T, Dehaen W, Clifford JN, Driesen K, Beljonne D, Van Averbeke B, Van der Auweraer M, Boens N (2007). J. Phys. Chem. A.

[72] Bozdemir OA, Guliyev R, Buyukcakir O, Selcuk S, Kolemen S, Gulseren G, Nalbantoglu T, Boyaci H, Akkaya EU (2010). J. Am. Chem. Soc.

[73] Qin W, Leen V, Rohand T, Dehaen W, Dedecker P, Van der Auweraer M, Robeyns K, Van Meervelt L, Beljonne D, Van Averbeke B, Clifford JN, Driesen K, Binnemans K, Boens N (2009). J. Phys. Chem. A.

[74] Qin W, Leen V, Dehaen W, Cui J, Xu C, Tang X, Liu W, Rohand T, Beljonne D, Averbeke BV, Clifford JN, Driesen K, Binnemans K, Auweraer MVD, Boens N (2009). J. Phys. Chem. C.

[75] Hoffmann K, Resch-Genger U, Mix R, Friedrich JF (2006). J. Fluoresc.

[76] Xiang S, Xing G, Xue W, Lu C, Lin J-M (2012). Analyst.

[77] Everhart DS, Reilley CN (1981). Anal. Chem.

[78] Graf N, Yegen E, Gross T, Lippitz A, Weigel W, Krakert S, Terfort A, Unger WES (2009). Surf. Sci.

[79] Yegen E, Zimmermann U, Unger WES, Braun T (2009). Plasma Processes Polym.

[80] Graf N, Yeğen E, Lippitz A, Treu D, Wirth T, Unger WES (2008). Surf. Interface Anal.

[81] Girard-Lauriault P-L, Dietrich PM, Gross T, Unger WES (2012). Surf. Interface Anal.

[82] Song X, Ma Y, Wang C, Dietrich PM, Unger WES, Luo Y (2012). J. Phys. Chem. C.

[83] Sugimura H, Moriguchi T, Kanda M, Sonobayashi Y, Nishimura HM, Ichii T, Murase K, Kazama S (2011). Chem. Commun.

[84] Moon JH, Kim JH, Kim K, Kang T-H, Kim B, Kim C-H, Hahn JH, Park JW (1997). Langmuir.

[85] Zhang F, Sautter K, Larsen AM, Findley DA, Davis RC, Samha H, Linford MR (2010). Langmuir.

[86] Imhof A, Megens M, Engelberts JJ, de Lang DTN, Sprik R, Vos WL (1999). J. Phys. Chem. B.

[87] Resch U, Rurack K (1997). Proc. SPIE.

[88] SAINT (1998). Bruker Analytical X-ray Instruments Inc..

[89] APEX2 (2001). Bruker Analytical X-ray Instruments Inc..

[90] Sheldrick GM (2002).

[91] Sheldrick GM (1997).

[92] Sheldrick GM (2008). Acta Crystallogr. Sect. A.

[93] (2004). Int. Org. f. Standardization.

[94] (2010). Int. Org. f. Standardization.

[95] Beamson G, Briggs D (1992). High Resolution XPS of Organic Polymers.

[96] Frisch MJ (2004). Gaussian, Inc..

