# Supplementary material for: Fluorinated Boron-Dipyrromethene (BODIPY) Dyes: Bright and Versatile Probes for Surface Analysis
Source: ChemistryOpen. 2013 Jan 9;2(1):25–38. doi: 10.1002/open.201200039 (PMC3594589; doi:10.1002/open.201200039)
Supplement: Supplementary file 1 [file open0002-0025-SD1.pdf]

ChemistryOPEN

Supporting Information

© 2013 The Authors. Published by Wiley-VCH Verlag GmbH & Co. KGaA, Weinheim

Fluorinated Boron-Dipyrromethene (BODIPY) Dyes: Bright and Versatile Probes for Surface Analysis

Mandy Hecht,[a] Tobias Fischer,[a] Paul Dietrich,[b] Werner Kraus,[c] Ana B. Descalzo,[a, d] Wolfgang E. S. Unger,[b] and Knut Rurack*[a]

open_201200039_sm_miscellaneous_information.pdf

Table of contents

Photostability measurements (incl. Fig. S1) S2
Table S1 Bond length and angles of the BF_2N_2 tetrahedron S3
Table S2 Additional selected spectroscopic data of **1**–**8** S4
Table S3 Important geometric features of the calculated structures of **3** and **4** S5
Table S4 Total formal XPS elemental composition S6
Figure S2 High resolution N 1s spectra S7
Figure S3 High resolution F 1s spectra S8
Figure S4 High resolution C 1s spectra S9
Figure S5 Fluorescence spectra of **5**-labelled slides S10

Photostability measurements

The photostability of dyes **2**, **4**, **5**, **7**, **8** and **10** was recorded using a ND:YVO_4 laser (Millennia, Spectra Physics) with a stabilized output power of 0.2 W (for **2**, **5**, **7** and **10**) as well as 3 W (for **4** and **8**; because the absorption of these dyes is considerably smaller at the irradiation wavelength, only ca. ~ 1/10th, the laser power was increased, to keep the results comparable), directed onto an ultra-micro fluorescence cuvette (Hellma, 105.251-QS) containing 45 µL of the dye in acetonitrile with the absorbance adjusted to 0,25 at 532 nm for all the dyes. The fluorescence was recorded with a fibre spectrometer (HR4000, Ocean Optics).

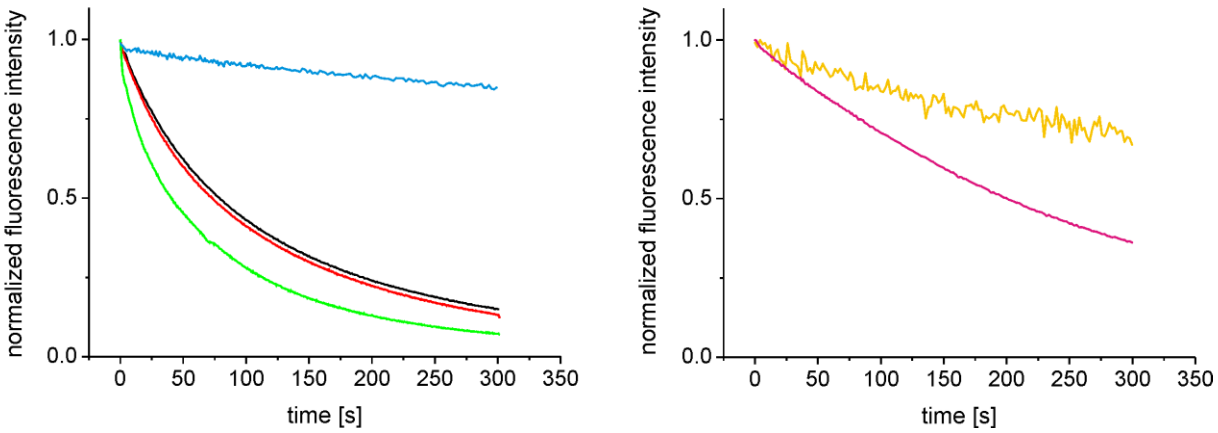

Figure S1. Decrease of fluorescence intensity of (left) **2** (black), **5** (blue), **7** (red) and **10** (green) and (right) **4** (yellow) and **8** (magenta) in acetonitrile as a function of time under cw-laser irradiation as described in the text.

Table S1. Bond lengths and angles of the BF_2N_2 tetrahedron.

Compound	B1-N1 [Å]	B1-N2 [Å]	B1-F1 [Å]	B1-F2 [Å]	N1-B1-N2 [°]	F1-B1-F2 [°]
**1**	1.556(4)	1.550(3)	1.384(2)	1.384(2)*	107.4(2)	110.5(2)*
**2**	1.556(5)	1.544(5)	1.397(4)	1.408(5)	108.1(3)	108.9(3)
**3**	1.551(4)	1.551(4)*	1.394(3)	1.394(3)*	108.0(4)*	110.2(4)*
**5**	1.568(6)	1.564(6)	1.378(5)	1.385(5)	106.1(3)	110.6(4)
	1.566(6)	1.581(6)	1.375(5)	1.386(5)	105.8(3)	111.5(3)
**6**	1.519(7)	1.579(8)	1.421(7)	1.365(7)	107.3(3)	109.8(4)
**7**	1.550(4)	1.529(4)	1.382(4)	1.388(4)	107.1(2)	110.0(2)
**8**	1.589(9)	1.564(9)	1.369(8)	1.424(8)	107.6(5)	110.6(6)

* Values resulting from symmetry operations

Table S2. Additional selected spectroscopic data of **1**–**8** in various solvents at 298 K.

Dye	Solvent[a]	λ_{abs} /nm	λ_{em} /nm	$\Delta\tilde{\nu}_{abs\text{-}em}$ /cm^{-1}	Φ_f	τ_f /ns	k_r[b] /10^8 s^{-1}	k_{nr}[b] /10^8 s^{-1}
**1**	Bu_2O	517	525	295	1.00	5.51	2.0	0.0
	Tol	521	531	361	1.00	4.98	2.3	0.0
	EtOH	515	524	334	1.00	5.88	1.7	0.0
	MeOH	514	523	335	1.00	6.12	1.7	0.0
**2**	Bu_2O	543	555	398	0.94	5.58	1.5	0.1
	Tol	547	560	456	0.96	6.23	1.7	0.1
	EtOH	541	554	434	0.90	6.53	1.4	0.2
	MeOH	540	553	435	0.89	6.69	1.3	0.2
**4**	Bu_2O	697	770	1360	0.17	1.04	1.6	8.0
**5**	Bu_2O	521	536	537	0.16	0.98	1.9	8.6
	Tol	525	544	665	0.38	1.96	1.9	3.2
	EtOH	518	534	578	0.14	0.88	1.6	9.8
	MeOH	516	532	583	0.14	0.93	1.5	9.3
**6**	Bu_2O	507	517	382	0.58	2.05	2.1	1.5
	Tol	510	521	451	0.88	3.59	2.5	0.3
	EtOH	505	516	422	0.47	2.35	2.0	2.3
	MeOH	504	514	424	0.48	2.59	1.9	2.0
**7**	Bu_2O	531	543	416	0.66	4.54	1.5	0.8
	Tol	534	549	512	0.70	4.96	1.4	0.6
	EtOH	530	545	553	0.66	4.41	1.5	0.8
	MeOH	529	543	487	0.65	4.85	1.3	0.7
**8**	Bu_2O	641	653	357	0.75	4.6	1.6	0.6
	Tol	647	660	350	0.75	4.42	1.7	0.6

[a] Hex = *n*-hexane, Bu_2O = dibutyl ether, Et_2O = diethyl ether, THF = tetrahydrofuran.
[b] $k_r = \Phi_f \times \tau_f^{-1}$, $k_{nr} = (1–\Phi_f) \times \tau_f^{-1}$; measurement uncertainties: ± 0.01 × 10^8 s^{-1}.

Table S3. Selected dihedral angles between various planes of the energy-minimized BODIPY structures of **3** and **4** as obtained by the density functional theory (DFT) method employing the hybrid functional B3LYP with a 6-31G basis set.

Compound	$\Theta_{py-py'}$[a] /Angle [°]	$\Theta_{dp-meso}$[b] /Angle [°]	$\Theta_{sty-sty'}$[c] /Angle [°]	Θ_{dp-sty}[d] /Angle [°]
**3**	2.8	77.3	20.2	23.7<br>23.7
**4**	2.0	76.9	33.0	48.8<br>34.8

[a] py, py' = planes defined by pyrrole rings of BODIPY core (C1–C4,N1 and C5–C9,N2), dp = plane defined by atoms C1–C9,N1,N2,B1 for dp and C10–C15 for *meso*-group, sty, sty' = planes defined by atoms of the aromatic parts of the styryl extensions.

Table S4. Total formal XPS elemental composition of investigated slides in at-%.

EC[a]	bare glass[b]	APTES[c]	APTES + **5**(I)[d]	APTES + **5**(II)[d]	APTES + **5**(II)[e]	**5**[f]
	A[g]	B[g]	C[g]	D[0] [g]	D[60] [g]	E[g]
C	6.3	51.0	38.6	43.2	50.6	8.9
N	0.5	7.4	6.5	7.0	8.3	1.1
O	63.5	28.2	31.6	27.7	21.1	64.3
Si	21.4	13.1	16.7	14.9	10.5	21.7
F	–	–	2.6	4.7	8.0	–
Na	4.7	0.3	1.8	1.3	1.2	1.5
Ca	1.6	–	–	–	–	0.9
Mg	1.2	–	1.4	0.8	–	0.8
Al	0.9	–	–	–	–	0.9
Zn	–	–	0.6	0.4	0.4	–

[a] EC = elemental composition and atom number ratios. [b] Piranha-cleaned slide. [c] APTES-functionalized slide. [d] Two different APTES-functionalized slides reacted with **5**. [e] Slide D observed at an electron emission angle of 60° instead of 0°; all other measurements also performed at 0°. [f] Piranha-cleaned slide (without APTES), treated with **5** and subjected to normal washing/drying procedure. [g] Slide abbreviation.

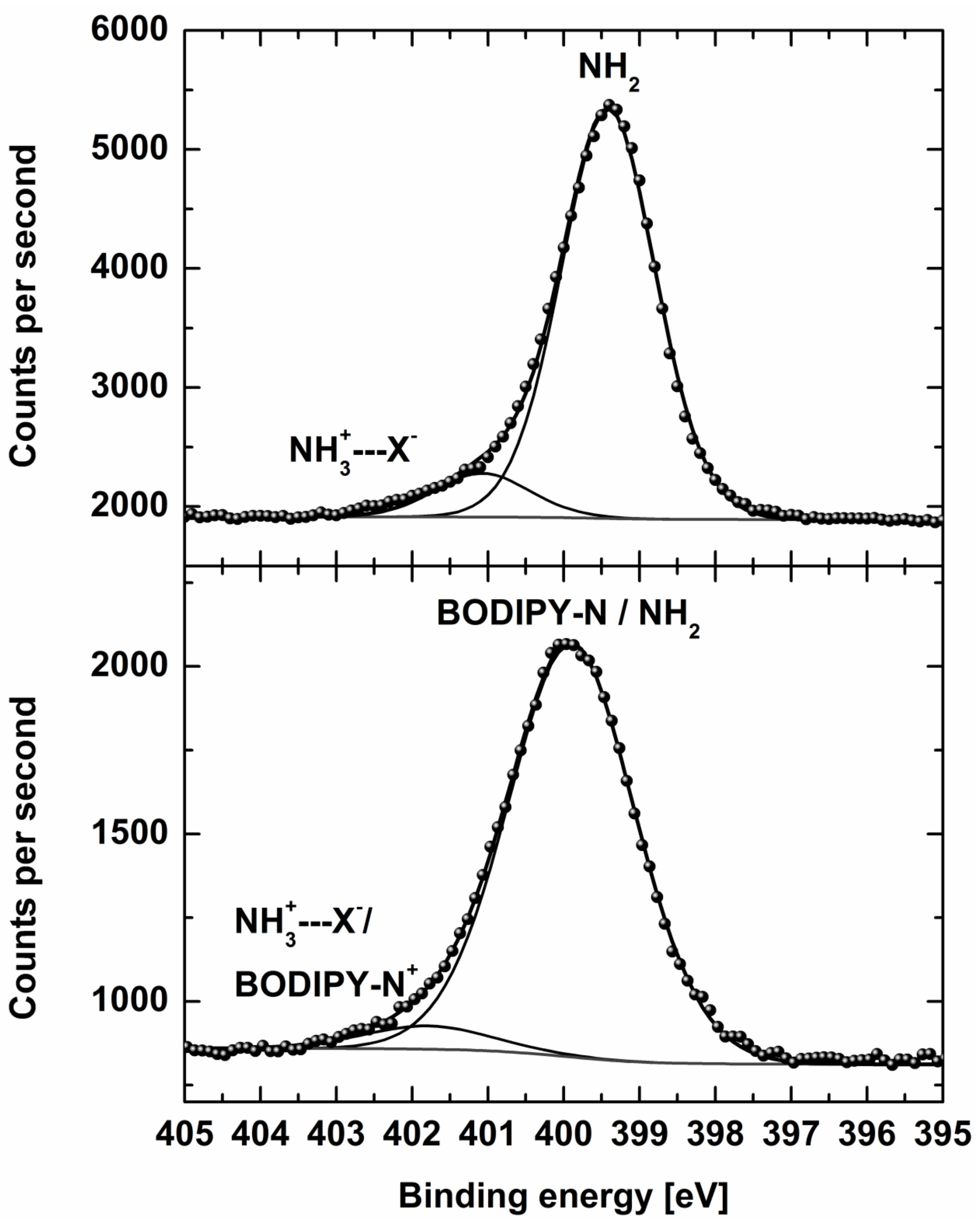

Figure S2. XPS high resolution N 1s spectra of the dye covered area of the slides shown in Figure 7, amino-functionalized slide prior to (slide B, top) and after reaction with **5** (slide D, bottom) as described in the main article.

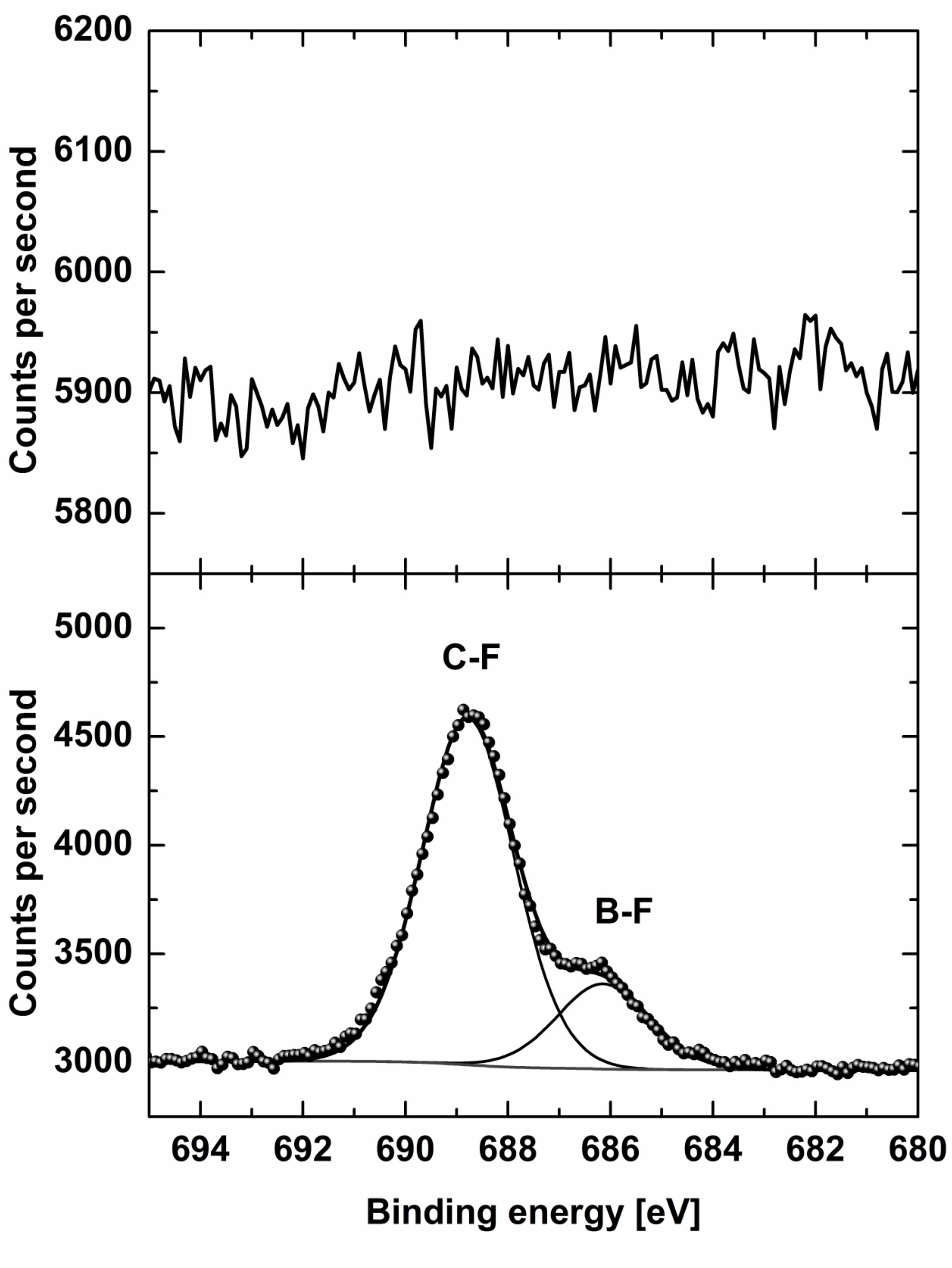

Figure S3. XPS high resolution F 1s spectra of the dye covered area of the slides shown in Figure 7, amino-functionalized slide prior to (slide B, top) and after reaction with **5** (slide D, bottom) as described in the main article.

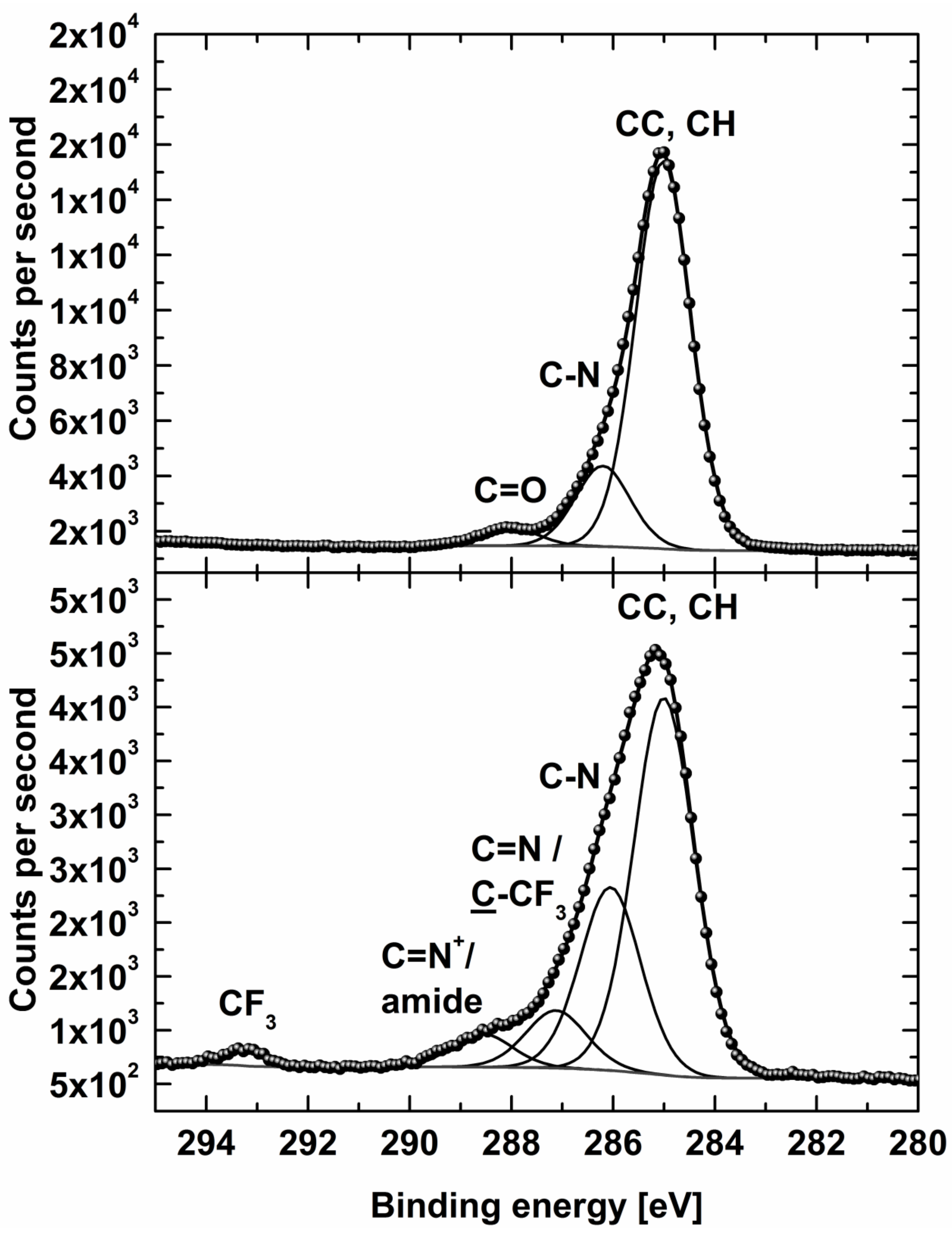

Figure S4. XPS high resolution C 1s spectra of the dye covered area of the slides shown in Figure 7, amino-functionalized slide prior to (slide B, top) and after reaction with **5** (slide D, bottom) as described in the main article.

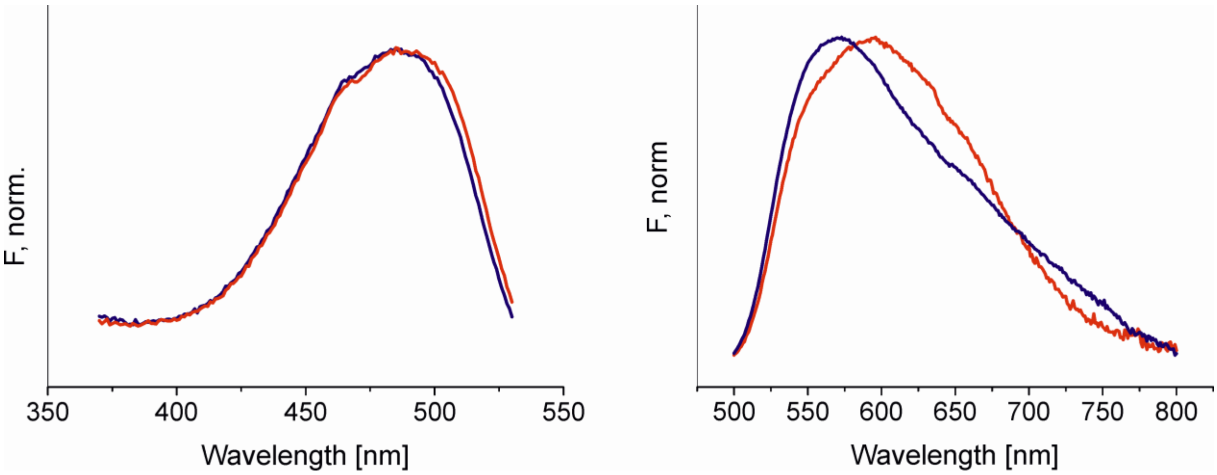

Figure S5. Fluorescence excitation (left, observed at 590 nm) and emission (right, excited at 466 nm) spectra of the two **5**-labelled slides discussed in the main text; slide C (blue) and slide D (red).
